# Efficient models for enhancing the link adaptation performance of LTE/LTE-A networks

**DOI:** 10.1186/s13638-022-02091-w

**Published:** 2022-02-02

**Authors:** Ali Abdulqader Bin-Salem, Tat-Chee Wan, Hamad Naeem, Mohammed Anbar, Sabri M. Hanshi, Abdellah Redjaimia

**Affiliations:** 1 School of Computer Science and Technology, Zhoukou Normal University, Zhoukou, 466001 Henan People’s Republic of China; 2 School of Computer Sciences, Universiti Sains Malaysia, 11800 Penang, Malaysia; 3 National Advanced IPv6 Centre, Universiti Sains Malaysia, 11800 Penang, Malaysia; 4 Seiyun Community College, Hadhramout, Yemen; 5 School of Computer Science, Neijiang Normal University, Neijiang, 641100 Sichuan People’s Republic of China

**Keywords:** Link adaptation, Modulation scheme, LTE/LTE-A networks, Cross-layer approach, Markov decision process

## Abstract

Link adaptation (LA) is the ability to adapt the modulation scheme (MS) and the coding rate of the error correction in accordance with the quality of the radio link. The MS plays an important role in enhancing the performance of LTE/LTE-A, which is typically dependent on the received signal to noise ratio (SNR). However, using the SNR to select the proper MSs is not enough given that adaptive MSs are sensitive to error. Meanwhile, non-optimal MS selection may seriously impair the system performance and hence degrades LA. In LTE/ LTE-A, the LA system must be designed and optimized in accordance with the characteristics of the physical (e.g., MSs) and MAC layers (e.g., Packet loss) to enhance the channel efficiency and throughput. Accordingly, this study proposes using two LA models to overcome the problem. The first model, named the cross-layer link adaptation (CLLA) model, is based on the downward cross-layer approach. This model is designed to overcome the accuracy issue of adaptive modulation in existing systems and improve the channel efficiency and throughput. The second model, named the Markov decision process over the CLLA (MDP-CLLA) model, is designed to improve on the selection of modulation levels. Besides that, our previous contribution, namely the modified alpha-Shannon capacity formula, is adopted as part of the MDP-CLLA model to enhance the link adaptation of LTE/LTE-A. The effectiveness of the proposed models is evaluated in terms of throughput and packet loss for different packet sizes using the MATLAB and Simulink environments for the single input single output (SISO) mode for transmissions over Rayleigh fading channels. In addition, phase productivity, which is defined as the multiplication of the total throughput for a specific modulation with the difference between adjacent modulation SNR threshold values, is used to determine the best model for specific packet sizes in addition to determine the optimal packet size for specific packet sizes among models. Results generally showed that the throughput improved from 87.5 to 89.6% for (QPSK $\rightarrow$ 16-QAM) and from 0 to 43.3% for (16-QAM $\rightarrow$ 64-QAM) modulation transitions, respectively, using the CLLA model when compared with the existing system. Moreover, the throughput using the MDP-CLLA model was improved by 87.5–88.6% and by 0–43.2% for the (QPSK $\rightarrow$ 16-QAM)and (16-QAM $\rightarrow$ 64-QAM) modulation transitions, respectively, when compared with the CLLA model and the existing system. Results were also validated for each model via the summation of the phase productivity for every modulation at specific packet sizes, followed by the application one-way analysis of variance (ANOVA) statistical analysis with a post hoc test, to prove that the MDP-CLLA model improves with best high efficiency than the CLLA model and the existing system.

## Introduction

In 2004, an initial study of long-term evolution (LTE) was introduced and viewed as a path for migration to 4G networks [1]. The goal of LTE is to increase the speed and capacity of wireless networks by utilizing signal processing techniques and modulations [2]. Long-term evolution-advanced (LTE-A) becomes the project that achieves the requirements of 4G technology.

LTE and LTE-A are the primary communication technology and have a capability for the deployment in a place where it becomes complicated to get with other technologies such as digital subscriber line (DSL) or cable. This complicated process is because of the deployment’s cost; maintenance of such technologies; and the distinguished features of LTE, such as the high capacity of transmission bandwidth that reaches to 20 and 100 MHz in LTE and LTE-A, respectively [3]. Such bandwidth will lead to raise the data rate up to 100 Mbps and 1000 Mbps for downlink (DL) LTE and DL LTE-A, respectively [4], and hence achieve good quality of service (QoS). However, given the extensive spreading of multimedia applications (i.e., voice, video), allowing the QoS in this scope becomes vital [5].

Recently, the data transferred by wireless network devices and multimedia system are merged and developed as one of the main parts for this mode of network. In view of the outbreak of the highly contagious COVID-19 pandemic, millions of people are now working and studying from home using online services and software such as movie streaming applications, video conferencing tools, and online schooling applications. The high demand for network bandwidth due to these services led to a degradation of broadband network performance across the board. Spillover effects are expected on wireless cellular networks even after the end of mandatory lockdowns due to the increased use of such services in the typical work and study environment. Despite the promise of 5G technology to provide higher bandwidth capacities to meet the increases in bandwidth demand, the slow-paced 5G-network deployment in many countries means that existing LTE/LTE-A networks will experience degraded network performance for the foreseeable future. As videos are streamed, the broadband wireless networks are influenced by many factors that negatively affect the quality. For example, the dynamic nature of wireless networks with user mobility poses challenges in maintaining the quality and transmission of video. In addition, distance has drastically affects the received signal strength (RSS), which then directly affects the quality of video transmission. As the outcome of this effect takes action, the channel efficiency and video quality are considerably reduced, which result in degrading the network performance [6, 7].

The LTE/LTE-A standards use frame aggregation in the MAC layer, that is, multiple numbers of MAC Service Data Unit (MSDUs) are transmitted via one frame transmission. Using a larger aggregated packet size in the MAC layer introduces a lower transmission reliability and higher channel efficiency and vice versa. This finding indicates that packet size could affect system performance. Thus, an improper packet size could negatively affect system performance.

LA is a technique used to adapt modulation to the link budget for improved adjustments of the changing channel conditions [8]. Meanwhile, LA techniques take into consideration channel conditions and the dynamic nature of broadband wireless networks. The LA technique presented in [9] uses the instantaneous channel state information (CSI) for controlling and adapting the data traffic of wireless channel. In addition, services such as higher data and lower packet error rates can be provided by the system adaptation. Typically, the spectral efficiency and maximized throughput are improved by adapting the modulation and coding (AMC) at the physical layer and subsequently including it in the LA technique. Furthermore, LA consists of frame error rate (FER) as performance factor at the link layer for reliability improvement of the system.

The proposed work aims to tackle the following challenges:

1. Reduction in channel efficiency and challenges of path loss that occur because of varying in channel conditions such as fast dynamic nature of wireless networks with user mobility and stringent requirements of QoS (required packet loss) [10, 11]
2. The inaccuracy of dynamic modulation switching in the existing approach due to the misprediction of switching levels, and unspecified policy for selecting the best MS for various link conditions.
3. Using improper packet size could introduces a lower transmission reliability and/or affects channel efficiency, and hence, it could negatively affect system performance as represented in [12].

The current proposed work extends our previous paper [13], which evaluated the performance of the existing LTE/LTE-A system in terms of throughput and packet loss for orthogonal frequency-division multiple access (OFDMA) modulation scheme with different packet sizes. The modified alpha-Shannon capacity formula addressed in [3] is adopted as part of the reward function to enhance the link adaptation of LTE/LTE-A. Furthermore, this paper extends the work presented in [14] by proposing a dynamic optimization framework for link adaptation that can maximize channel efficiency and throughput for LTE/LTE-A; moreover, it is used to design the reward function and transition probability. It is different from [14] owing to its adoption of the modified alpha-Shannon capacity formula in MDP in addition to implement this work in the LTE/LTE-A environment rather than WiMAX. Besides that, parameters such as bandwidth efficiency factor $\alpha$, target-required SNR factor $\omega$, and modulation value, that will be discussed in the proposed model section, are not parts of the previous work. The proposed work in this paper enhances the optimal decision of link adaptation over LTE/LTE-A networks and fits their quality requirements.

The contribution of this research can be summarized as follows:

1. The CLLA model is proposed, which adapts several parameters across physical and MAC layers including the analytical prediction of packet loss for the next modulation for improvement in throughput and adaptive modulation.
2. Because of the limitation accuracy issue of adaptive modulation in CLLA model, the dynamic optimization MDP-CLLA model is proposed, which predicts the switching levels of adaptive modulation scheme more precisely and hence selects the optimal modulation level more accurately and offers more enhancement in throughput and LA of LTE/LTE-A.
3. The performance (throughput, packet loss, and phase productivity) of proposed framework is evaluated against the existing approach with respect to the variation of different packet sizes and modulation schemes. The evaluation and comparison with the existing approach confirm the feasibility of the proposed models. Furthermore, the suggested models have been evaluated in terms of optimal packet size, which shows that the proposed work has a significant impact on the phase productivity when compared to the existing work.

Many researchers [10, 11, 15–17], by utilizing various methods, contribute to the LA area. Such methods (techniques) are cluster-based channel envelope and phase predictor for broadband wireless systems, cross-layer architecture, Markov chain, outer loop link adaptation (OLLA), and contextual multi-armed bandits (MAB). However, there are still gaps and more effort is needed to address them. The related work section explains these existing projects in depth.

The rest of the paper is organized as follows: Sect. 2 provides an overview about LTE/LTE-A network, the significance of LA, and the channel quality indicator metrics required for this study. Section 3 discusses the related work for enhancing the LA of previous researchers and their limitation. Section 4 mathematically determines and validates the proposed models’ preliminaries such as PER and SNR threshold values for different modulation types with different packet sizes. Furthermore, the proposed CLLA and MDP-CLLA models including the formulation are presented in Sects. 5 and 6, respectively. In Sect. 7, the evaluation criteria used for measuring the performance of proposed models are highlighted. Section 8 presents the simulation environment used for the existing LTE/LTE-A system and for applying the proposed models. Results and discussion for throughput, packet loss, and overhead packet size (phase productivity) that show the optimal packet size and the best phase productivity among models are presented and discussed in Sect. 9. In addition, Sect. 10 further discusses the result validation. Finally, Sect. 11 concludes this paper.

## Background

This section provides an overview about LTE/LTE-A networks, the importance of LA, and the channel quality indicator measures required for this study.

### LTE/LTE-A

LTE and LTE-A play a significant role in offering connectivity to the Internet. However, with the widespread of multimedia applications, the demand for QoS support (i.e., bandwidth transmission) in LTE and LTE-A has been increased dramatically. The bandwidth transmission for LTE and LTE-A can cover up to 5 km of urban and suburban areas and up to 100 km in rural areas. Because of these characteristics, LTE/LTE-A is a suitable option, offering cost-efficient and quick to be deployed. The LTE/LTE-A deployment architecture utilized in this research is point-to-point (PTP) since it aims to improve video transmission by taking the dynamic nature of broadband wireless networks and adjusting modulation based on the signals received and SNR measurements [18]. LTE/LTE-A provides a variety of modulations that may get complex when data rates are large. Therefore, decoding, in this case, requires a specific degree of SNR while keeping noise sensitivity into account. Meanwhile, the modulations that provide simple low data rates have a wide scope. Thus, a low data rate requires less powerful signals to decode which leads to extending the scope of coverage of a wireless network at low data rates [19]. Furthermore, modulations can be integrated with optimal packet size and other upper-layer factors to enhance link adaptation to improve the performance of throughput and packet loss in the network.

### The importance of LA

The importance of LA is coming from that, in data and communication networks, the transmitted data are treated with unequal importance especially for video content and the loss of some video packets has a higher distortion impact on the received video quality compared to other less important packets, in addition to the difficulties of keeping growing the data rate as high as possible within bandwidth and power restrictions. To overcome this issue, spectral efficiency must be driven to the maximum [20]. Wireless channels are time-varying and frequency-selective in nature. Therefore, LA can be used to better utilize instantaneous capacity through adjusting modulations. In LA modulations, the device must constantly measure and report SNR or SINR experienced at the receiving device in order for the network to make a decision on the suitable downlink modulations. In addition, a quality channel report (channel characteristics) is required from the network in order to update the LA through estimating the effects of a switch to another modulation. In other words, the network may determine when to seek a report and how frequently [21]. Devices that use LA are applied in many industrial fields such as military, aircraft, communications, healthcare, factories, and education. AeroMACS is one of the systems that are used in the airport channel and it depends mainly on LA for adapting modulation and achieving high spectral efficiency while maintaining received signal reliability at an acceptable level [22]. A lot of studies have been conducted in LA to further enhance the channel efficiency, and common studies are thoroughly explained in the related work section.

### Channel quality indicator metrics

The main parameter influencing QoS parameters, especially throughput and packet loss, is SNR, which is the ratio between the maximum signal strength that a wireless connection can achieve and the noise present in the connection [23]. When the throughput adaptation is applied in the downlink channel, the current throughput becomes dependent on SNR. Furthermore, the BER approximation of modulation schemes has an important effect on throughput. Understanding the relationship between SNR and modulation requires us to know the concept of modulation. Modulation is the process of carrying a message signal over another signal that can be physically transmitted. Several kinds of modulation and coding rates are available in LTE/LTE-A with varying physical speeds from 1 Mbps to 1 Gbps in recent standards and up to 3 Gbps in multiple-input-multiple-output (MIMO). A high data rate uses complicated modulations that allocate more bits in a time interval than a low data rate. These complicated modulations are sensitive to noise and require a certain level of SNR for decoding. LTE/LTE-A allows the modulation scheme to change on a burst-by-burst basis per link based on channel conditions. For the downlink, unit equipment (UE) provides eNodeB with feedback on the downlink channel quality by using a channel quality indicator (CQI). For the uplink, eNodeB can estimate channel quality on the basis of the received signal quality. The eNodeB scheduler assigns a modulation and coding scheme for the available SNR to maximize the throughput on the basis of the channel quality of each user’s uplink and downlink. Thus, AMC increases the overall system capacity.

## Related work

In the literature, several researchers have proposed different methods for the improvement of LTE/LTE-A networks’ performance. For example, methods including link adaptation, power allocation, and QoS requirements were proposed. In [15], for example, a new cluster-based channel envelope and phase predictor for broadband wireless systems such as LTE was proposed. Such predictor operates in the time domain on each modulation coding. In addition, implementing the channel envelope predictor as a linear predictor is simple but sensitive to estimation error. Regularly, separation of estimation error is performed by operating a narrow band-pass filter in the time domain for each modulation coding (MC). The work in [15] shows the prediction accuracy in low channel SNR, which can improve link adaptation but does not consider the accepted packet error rate (PER) that may affect the performance of the link adaptation. Researchers in [16] developed distributed cross-layer architecture for network awareness and opportunistic transport (DISCO) to enable network awareness and adapt transport, network, and application parameters in mobile ad hoc networks (MANET). As presented, the approach can better meet applications’ performance requirements and improve throughput. This architecture uses TCP-Derwood at a transport layer, which provides remarkable performance on link switching. Nevertheless, the testing [16] is performed on the basis of a successful deliver file size at the destination not based on streaming. In addition, AMC and PER that notably influence link adaptation are not considered in their work. In the case of adopting Markov decision process (MDP) over cross-layer, the discrete-time Markov chain over cross-layer design is proposed in [17] as a cognitive content delivery system to provide best-effort access to recommended contents tailored to the real-time traffic condition. It incorporates physical, MAC, and application layers. The finding of the method in [17] shows that a trade-off exists between increasing throughput and decreasing delay that affects the content delivery. However, their evaluation considers the file size but not the streaming [17]. Researchers in [10] developed a new framework for AMC under block error constraints through logistic regression modeling for the BLER. In addition, a sub-optimal procedure with a single optimization parameter is also highlighted and added to the framework. Later, an outer loop link adaptation (OLLA) technique is added as an optimizer converging to this set of sub-optimal thresholds. However, although OLLA converges in average to the sub-optimal solution, it is impractical because OLLA does not meet to a single solution but wanders over the set of thresholds that can keep the BLER within an accepted range [10]. Moreover, OLLA suffers from slow convergence and finally degrades the average link throughput [10, 24]. In [11], an online LA for selecting the optimal MCS to increase throughput in cellular communication systems is proposed. It relies on contextual multi-armed bandits (MAB) technique and an artificial neural network (ANN) model to predict the transmission success probability for each MCS given a link context vector that contains the reported channel state and additional link side information. However, the work is still not an optimal solution because it fails to consider channel transition probability and hence leads to sub-optimal link throughput. In addition, it ignores the need to determine a target error rate, which leads to a negative significant impact on LA. In nutshell, there is a substantial effort required to improve the performance of link adaption. This is due to that the existing research work suffers the following limitations (i) reduction in channel efficiency and issue of path loss due to varying in channel conditions, (ii) the inaccuracy of dynamic modulation switching, (iii) susceptibility to error, (iv) target error rate, (v) improper packet size.

## Proposed models’ preliminaries

Before discussing the proposed models, the PER and SNR threshold values for several packet sizes are determined in this section.

### PER

To avoid confusion of symbol or bit, the latter is used as the representative in packet size and error rate rather than symbol in this paper. Therefore, PER is determined using BER and the bits’ numbers in the packet. The packet’s length in bits is denoted by *L*, and the bit error probability for the channel is denoted by $P_{e}$. If the error of the packet exceeds the threshold value of BER, then it will be ignored. According to [25], the PER can be computed as follows:

1 $$\begin{aligned} PER=1-(1-P_{e}(\gamma ))^{L} \end{aligned}$$

As a packet, the PER must not exceed 10%, hence:

2 $$\begin{aligned} PER\le 0.1 \end{aligned}$$

Therefore, 90% of the transmitted packets should be received correctly, hence:

3 $$\begin{aligned} P_{\mathrm{accept}}\ge 0.9 \end{aligned}$$

$P_{\mathrm{accept}}$ can be expressed as:

4 $$\begin{aligned} P_{\mathrm{accept}}\ge (1-\mathrm{BER}_{\max })^{L} \end{aligned}$$

The amount of BER that corresponding to 10% of PER can be derived through substituting equation (3) in Eq. (4) as:

5 $$\begin{aligned}&0.9^{1/L}\ge 1-\mathrm{BER}_{\max } \end{aligned}$$

6 $$\begin{aligned}&\mathrm{BER}_{\max }=1-0.9^{1/L} \end{aligned}$$

For instance let L=8000 then:

7 $$\begin{aligned} \mathrm{BER}_{\max }=1.3*10^{-5} \end{aligned}$$

Hence, for receiving packet within the acceptable error:

8 $$\begin{aligned}&\mathrm{BER}\le \mathrm{BER}_{\max } \end{aligned}$$

9 $$\begin{aligned}&\mathrm{BER}\le 1.3*10^{-5} \end{aligned}$$

Table 1 represents the target 10% PER for different packet sizes.

**Table 1:** The corresponding BER values of 10% PER for various packet sizes

Packet size in bits	Maximum accepted BER of 10% PER
1000	$10^{-4}$
2000	$5.27\times 10^{-5}$
4000	$2.6\times 10^{-5}$
8000	$1.3\times 10^{-5}$

### SNR threshold values

Based on equation $\mathrm{BER}=0.2e^{-1.5\gamma _{\mathrm{tresh}}/(M-1)}$, the threshold values of SNR at Rayleigh fading channel could be specified as follows:

10 $$\begin{aligned}&\mathrm{BER}=\int _{0}^{\infty } P_{e}(\gamma )p(\gamma )\mathrm{d}\gamma \end{aligned}$$

11 $$\begin{aligned}&\mathrm{BER}=\int _{0}^{\infty }0.2e^\frac{{-1.5\gamma }}{M-1} (1/\gamma _{\mathrm{tresh}} exp(-\gamma /\gamma _{\mathrm{tresh}} ))\mathrm{d}\gamma \end{aligned}$$

12 $$\begin{aligned}&\mathrm{BER}=\frac{0.2}{\gamma _{\mathrm{tresh}} }\int _{0}^{\infty }e^{\frac{-1.5\gamma }{M-1}-\frac{\gamma }{\gamma _{\mathrm{tresh}} } }\mathrm{d}\gamma \end{aligned}$$

13 $$\begin{aligned}&\mathrm{BER}=\frac{0.2}{\gamma _{\mathrm{tresh}} }\int _{0}^{\infty }e^{-\gamma (\frac{1.5}{M-1}+\frac{1}{\gamma _{\mathrm{tresh}} })}\mathrm{d}\gamma \end{aligned}$$

14 $$\begin{aligned}&\mathrm{BER}=\frac{0.2}{\gamma _{\mathrm{tresh}} } \left[ \left( \frac{-1}{\frac{1.5}{M-1}+\frac{1}{\gamma _{\mathrm{tresh}} }}\right) e^{-\gamma \left( \frac{1.5}{M-1}+\frac{1}{\gamma _{\mathrm{tresh}} }\right) }\Big |_0^\infty \right] \end{aligned}$$

15 $$\begin{aligned}&\mathrm{BER}=\frac{0.2}{\gamma _{\mathrm{tresh}} } * -\left( \frac{\gamma _{\mathrm{tresh}} (M-1)}{1.5\gamma _{\mathrm{tresh}}+M-1 }\right) \left[ e^{-\gamma \left( \frac{1.5}{M-1}+\frac{1}{\gamma _{\mathrm{tresh}} }\right) }\Big |_0^\infty \right] \end{aligned}$$

Substituting $\gamma$ with $\infty$ and then 0, the equation will be:

16 $$\begin{aligned}&\mathrm{BER}=\frac{0.2}{\gamma _{\mathrm{tresh}} } * -\left( \frac{\gamma _{\mathrm{tresh}} (M-1)}{1.5\gamma _{\mathrm{tresh}}+M-1 }\right) \left[ 0-1 \right] \end{aligned}$$

17 $$\begin{aligned}&\mathrm{BER}= \frac{0.2(M-1)}{1.5\gamma _{\mathrm{tresh}}+M-1 } \end{aligned}$$

As BER is given, $\gamma _{\mathrm{tresh}}$ will be:

18 $$\begin{aligned}&1.5\gamma _{\mathrm{tresh}}=\frac{0.2(M-1)-\mathrm{BER}(M-1)}{\mathrm{BER}} \end{aligned}$$

19 $$\begin{aligned}&\gamma _{\mathrm{tresh}}=\frac{(M-1)(0.2-\mathrm{BER})}{1.5\mathrm{BER}} \end{aligned}$$

After convert watt to d*B*, the $\gamma _{\mathrm{tresh}}$ will be:

20 $$\begin{aligned} \gamma _{\mathrm{tresh}}=10*\log _{10}\frac{(M-1)(0.2-\mathrm{BER}))}{1.5\mathrm{BER}} \end{aligned}$$

Mathematically, modulation type and BER are factors that determine the SNR threshold values. The resulting SNR threshold values from Eq. (20) are validated by running the simulation with various initial seeds to ensure the variety of noises. At the end, the estimated average SNR threshold values are determined for various sizes of packet (see Table 1) as represented in Table 2. The aim of this experiment is to

- show and measure the SNR results mathematically and experimentally and the level of closeness from one another.
- use the simulation results of SNR threshold values to obtain the throughput and packet loss for various packet sizes with different modulation types.

Table 2 presents that the results of mathematical and practical SNR threshold values are close to one another. For the practical results, these values are slightly high in the case that the packet size is 8000 bits. This change is explained as follows: When an increment in packet size occurs, decrements are expected in the total packet numbers of the frame; hence, the probability of PER will be high and reach to 10%. Consequently, a slightly higher value of SNR is required to guarantee that the PER of packets is in the acceptable range.

**Table 2:** Theoretical and practical SNR values for different packet sizes

Packet size	Modulation type					
	QPSK		16-QAM		64-QAM	
	Theoretical SNR	Measurement SNR	Theoretical SNR	Measurement SNR	Theoretical SNR	Measurement SNR
1000	35.79	36.34	42.78	43.30	49	49.25
2000	38.8	39.99	45.79	46.78	52.02	53.21
4000	41.87	43.3	48.86	50.15	55.09	56.5
8000	44.84	49.34	51.8	56.2	58.05	58.05

## The proposed models

This paper extends the work presented in [14] by proposing a dynamic optimization framework for link adaptation that can maximize channel efficiency and throughput for LTE/LTE-A; it is also used to design the reward function and transition probability. It is different from [14] through that the previous work does not include the adoption of the modified alpha-Shannon capacity formula in MDP in addition to use the WiMAX rather than LTE/LTE-A environment used in this research. Moreover, parameters such as bandwidth efficiency factor $\alpha$, target-required SNR factor $\omega$, and modulation value, that are discussed in the next subsections, are not parts of the previous work. Nonetheless, they played an important role when they are added to this research.

The proposed work enhances the optimal decision of link adaptation for LTE/LTE-A and fits the quality requirements for the LTE/LTE-A. In fact, two link adaptation models are proposed. The first model is called cross-layer link adaptation (CLLA) and is based on the downward cross-layer design approach. The purpose is to consider the disparate of the system at MAC/physical layers. Consequently, at the physical layer, the received frame is measured on the basis of channel condition adaptation and path loss. It is measured by utilizing the mobility distance while adapting the PER at the MAC layer. However, the second model adopts the MDP over the cross-layer design approach (MDP-CLLA). In fact, the MDP-CLLA model uses the measured process of the CLLA model, where the selection of appropriate modulation is optimally chosen for the next frame and is sent as feedback to the sender. Additionally, the evaluation and comparison between the performance of proposed models and the existing LTE system are performed in terms of throughput, packet loss, overhead packet size (optimal packet size), and phase productivity for different modulation schemes with different packet sizes.

### CLLA model

**Fig. 1:**
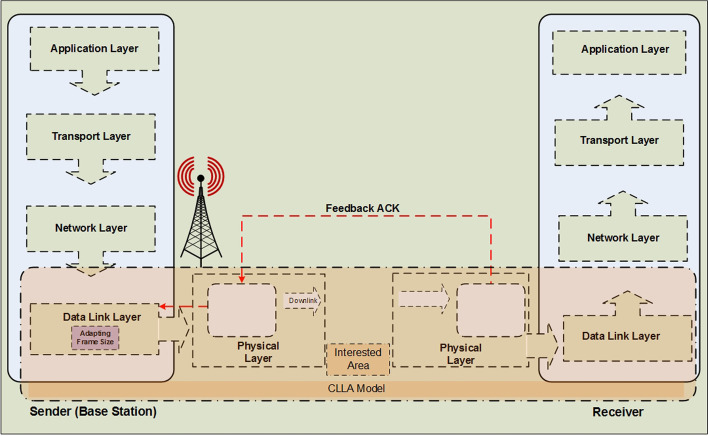
Framework of the proposed CLLA model

The framework of the CLLA model is illustrated in Fig. 1. It is considered a MAC/physical downward cross-layer design. At the receiver side, the SNR is measured at the physical layer by adapting a channel condition and path loss via a mobility distance parameter. Its purpose is to match transmission rates to a time-varying channel at the physical layer. In addition, the PER is measured and used as performance indicator at the MAC layer to improve reliability of the system. At the end, the suitable modulation type is selected, and a feedback is sent to the sender to be used as a trigger for adapting the modulation of the next transmission frame.

The algorithm of the CLLA model constitutes four parts: the main algorithm of the CLLA model; the packetization of data as indicator of MAC layer located at the transmitter side; the de-packetization of data at the receiver side; and the computing part of PER, number of error packets, and throughput. Algorithm 1 represents the algorithm of the CLLA model. 
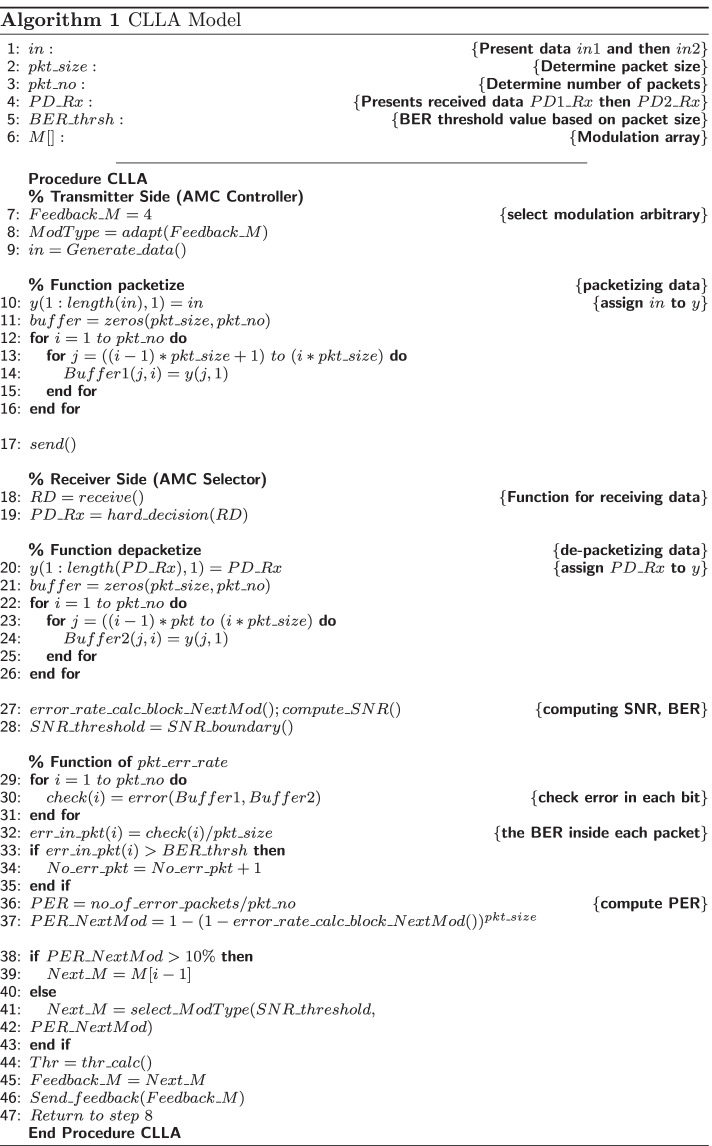


### Adopting the MDP over the CLLA model (MDP-CLLA)

**Fig. 2:**
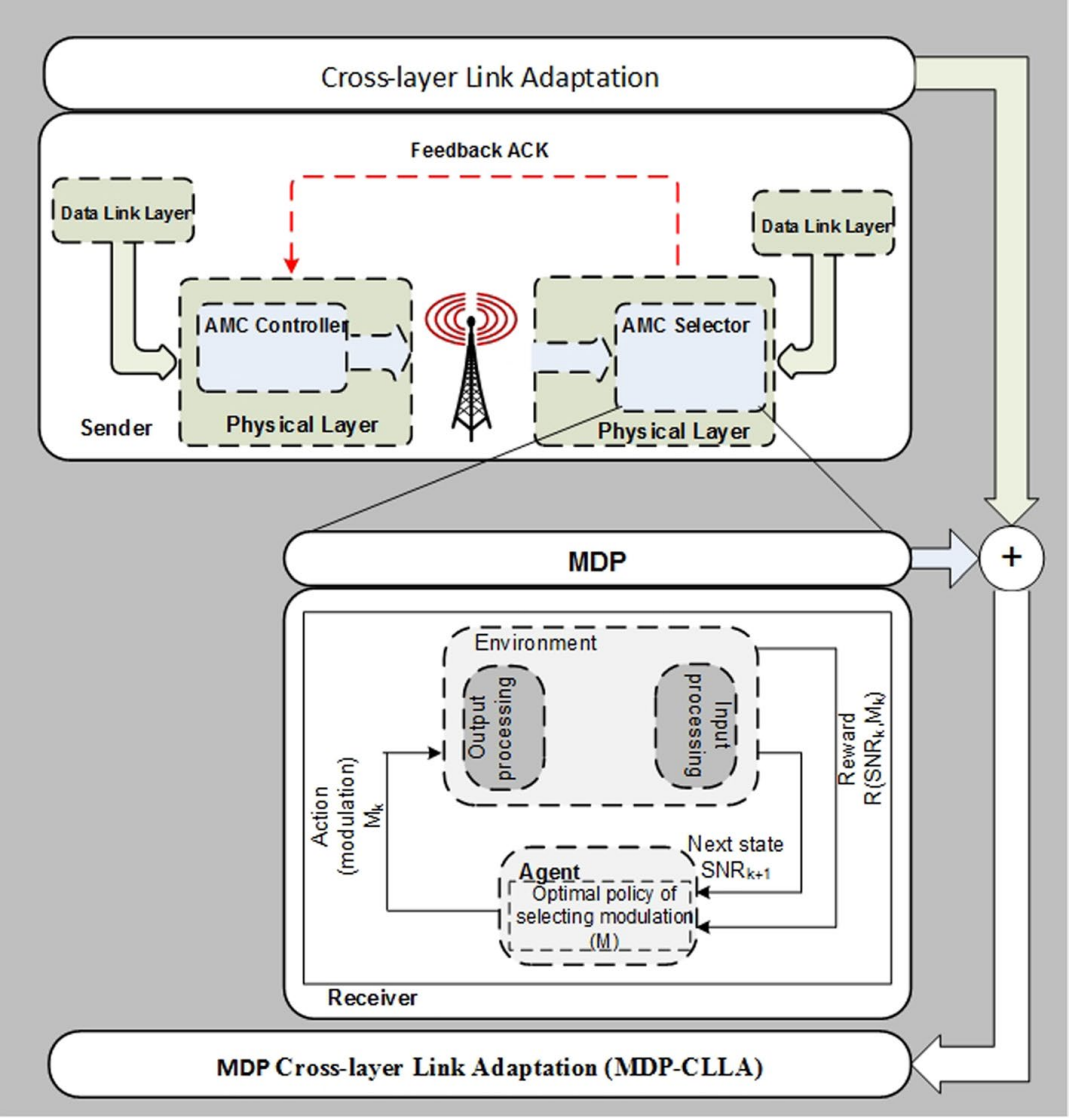
Framework of the proposed MDP-CLLA model

As shown in Fig. 2, the MDP-CLLA model uses MDP to the sequence procedure discussed in the CLLA model; hence, the utility/reward function of data transmission based on MAC/physical downward cross-layer technique is formulated systematically.

The MDP-CLLA model consists of modified alpha-Shannon capacity formula and link adaptation and reception scheme.

The modified alpha-Shannon capacity formula addressed in [3] is adopted as part of the reward function to enhance the link adaptation of LTE/LTE-A. The modified alpha-Shannon capacity formula was proposed to predict the LTE/LTE-A throughput accurately because of the implementation issues that face Shannon capacity bound and render it inapplicable to LTE/LTE-A. Such issues are system overhead (system level bandwidth efficiency factor) and implementation margins (SNR efficiency factor) such as channel estimation and CQI. In [26], a simulating model function of LTE/LTE-A evolved RAN is built for handling certain types of traffic in a vehicular network. It modifies the Shannon formula purely on the basis of the LTE/LTE-A bandwidth efficiency factor to fit the Shannon capacity to LTE/LTE-A, which is inaccurate because it overlooks the handling of the SNR efficiency factor. In [27], the alpha-Shannon capacity formula was proposed by modifying the Shannon formula through the addition of the LTE/LTE-A bandwidth efficiency factor and SNR efficiency factor. However, this formula is inaccurate in terms of the maximum capacity/throughput of standard LTE/LTE-A due to the test-bed that was done in [3]. Therefore, the modified alpha-Shannon capacity formula was proposed and discussed in [3] and is considered a part of this research.

**Fig. 3:**
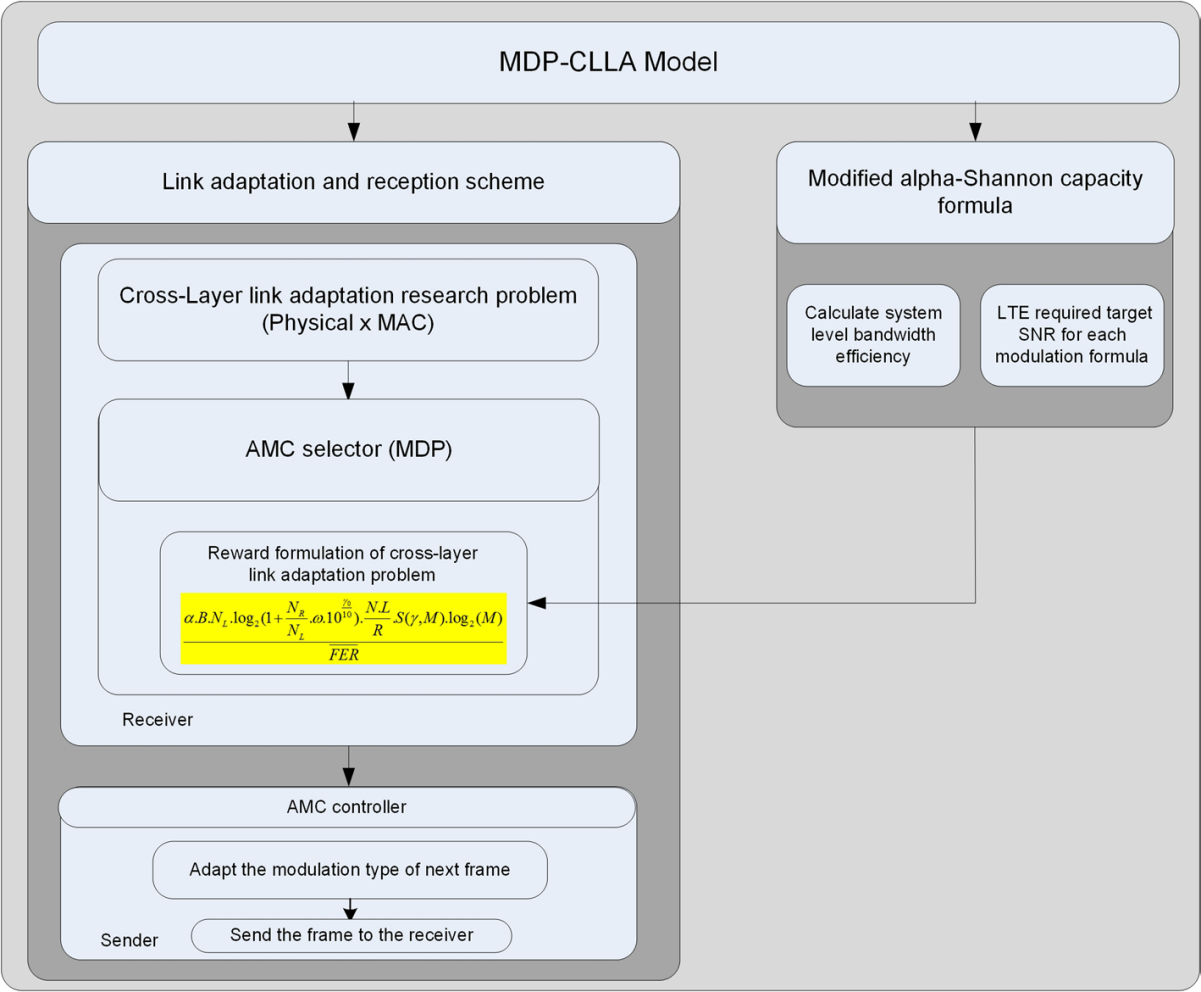
Proposed MDP-CLLA optimization model

Figure 3 thoroughly illustrates the proposed MDP-CLLA optimization model. In this proposed model, the average channel efficiency (throughput) maximization per FER minimization is studied considering the parameters of modulation, received power, mobility distance, channel condition, and path loss. The link adaptation and reception scheme in the MDP-CLLA model consists of AMC selector at the receiver side and AMC controller at the sender side as shown in Figs. 2 and 3. The AMC selector contains the MDP adopted over CLLA to overcome the optimal selection of modulation type. The AMC controller receives the feedback from the receiver and adapts the modulation type on the basis of this feedback. Then, this information is used to send the next frame. In addition, the proposed model is applied for different packet sizes to determine the appropriate packet size for each modulation.

**Fig. 4:**
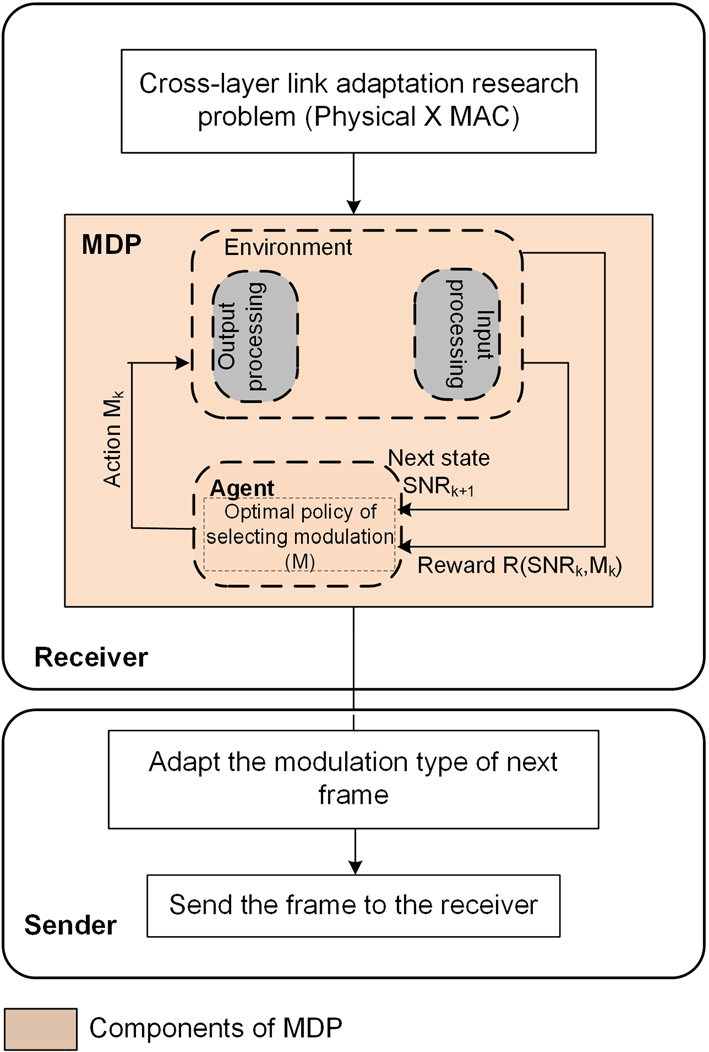
Process flow of proposed MDP-CLLA link adaptation and reception scheme

On the basis of MDP algorithm, Fig. 4 presents the process flow of MDP-CLLA link adaptation and reception scheme including the following components of MDP algorithm: states, actions, transmission probability, and reward function.

**Fig. 5:**
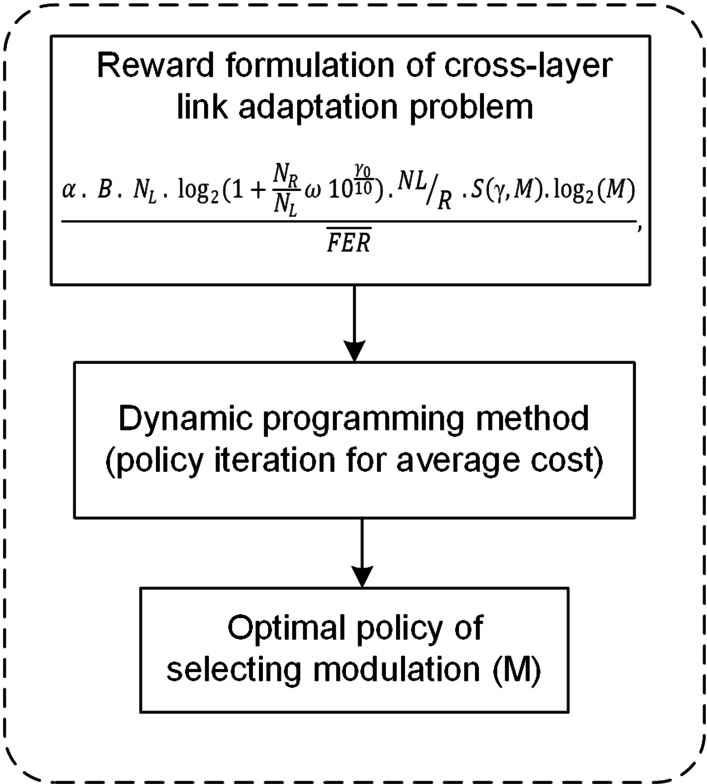
Dynamic programming-based MDP algorithm for optimal policy resulted by agent component

In addition, the MDP algorithm is solved by the agent component (see Fig. 4) through the use of policy iteration for average cost as a dynamic programming method as shown in Fig. 5.

The algorithm of the MDP-CLLA model consists of the main algorithm of MDP-CLLA model; the packetization of data at the transmitter side; the de-packetization of data at the receiver side; and the computing part of PER, number of error packets, and throughput as shown in Algorithm 2.

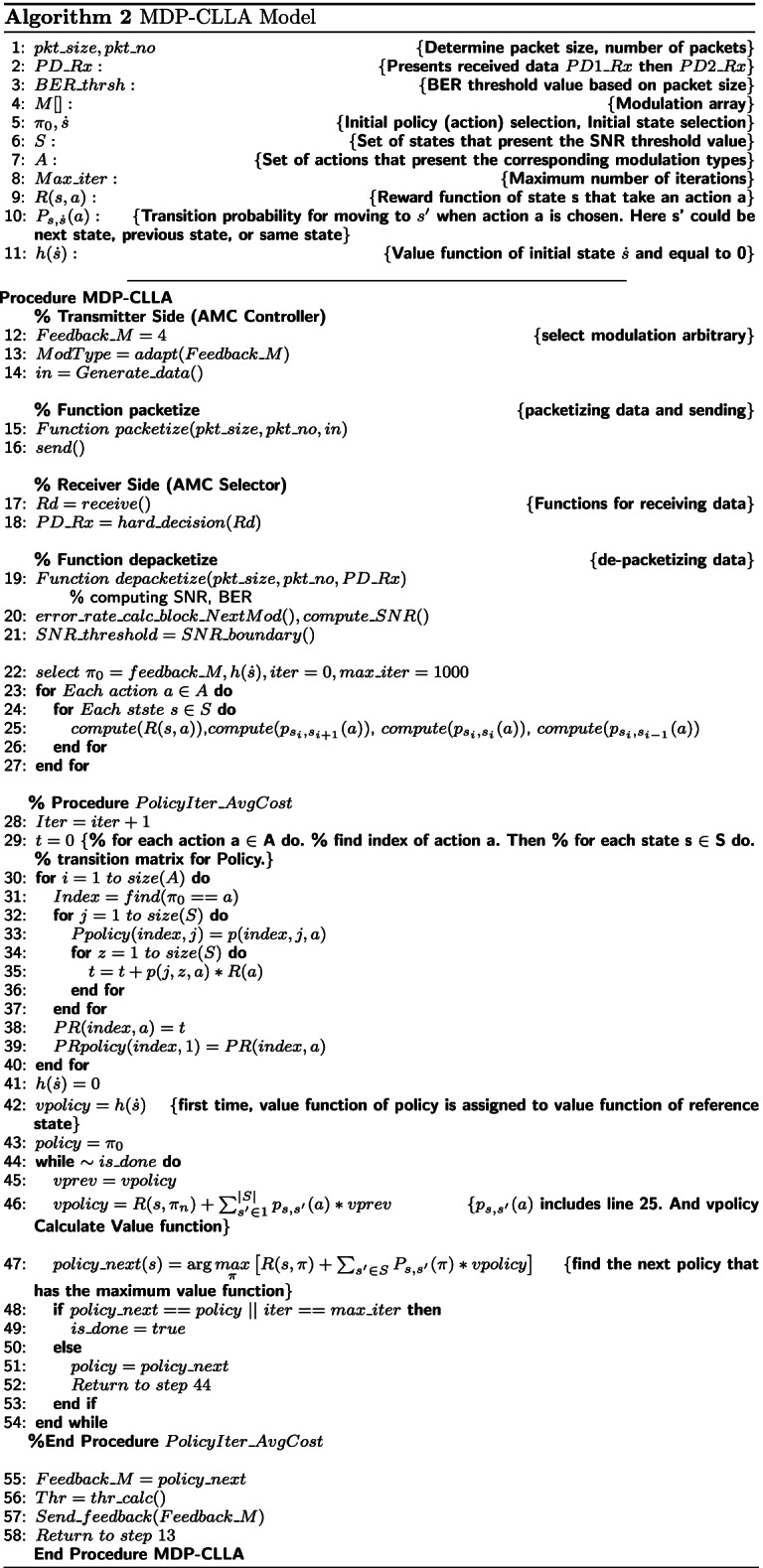



The reward function and the dynamic programming method used to obtain the optimal policy through the transition probability are presented below:

#### Reward function

Several studies on link adaptation have been discussed by using the reward function for throughput maximization. In [28], varying the rate of power transmission for each frame leads to maximize throughput. However, this work is based on changing the distance of user mobility, thereby affecting the calculation of SNR that enhances the throughput. Studying the enhancing throughput leads our concern on the transmission errors’ effects, which are not covered in the literature [29].

To increase the throughput, the range of FER must be adequate. Hence, increasing throughput per decreasing FER is considered the objective function. The proposed objective function is organized as follows:

1. The received frame comprises *NL* bits at the receiver side. Here, *N* is the number of packets in a frame, and *L* is the number of bits per packet. The current throughput becomes dependent of SNR as long as throughput adaptation is implemented in the downlink channel. Throughput can be calculated using the formula below [3]:
  21 $$\begin{aligned} \alpha *B*\log _{2}\left( 1+\omega *10^{\frac{\mathrm{SINR}_{\mathrm{d}B}}{10}}\right) \end{aligned}$$
  where $\alpha$ is a bandwidth efficiency factor, B is a bandwidth, and $\omega$ is a value related to target-required SNR, which is proven mathematically in [3].
2. The frame is considered the data unit transmitted in point-to-point communication system. Therefore, the frame period time for each transmission is $\frac{NL}{R}$ sec, where *R* is the transmission rate. The frame throughput (*Th*) is given as follows:
  22 $$\begin{aligned} \alpha *B*\log _{2}\left( 1+\omega *10^{\frac{\mathrm{SINR}_{\mathrm{d}B}}{10}}\right) *\frac{NL}{R} \end{aligned}$$
3. In addition, the number of bits for each modulation type must be added to the objective function, and it is done by $\log _{2}(M)$.
  23 $$\begin{aligned} \alpha *B*\log _{2}\left(1+\omega *10^{\frac{\mathrm{SINR}_{\mathrm{d}B}}{10}}\right)*\frac{NL}{R}*\log _{2}(M) \end{aligned}$$
4. Now, to calculate the average frame error rate ${\overline{\mathrm{FER}}}$ as in the proposed objective function:
  Let PER imply packet error rate $1-(1-P_{e})^{L}$. Thus, the FER is given as follows:
    24 $$\begin{aligned} \mathrm{FER}=1-(1-P_{e})^{NL} \end{aligned}$$
  Since FER should be within an acceptable range, SNR $\gamma$ is partitioned into K intervals with boundary points denoted as $0=\Gamma _{0}< \Gamma _{1}<\cdots <\Gamma _{k}$. Hence, mode k is selected when $\Gamma _{k}< \gamma <\Gamma _{k+1}$.
  FER, when using the $k_{th}$ mode, is denoted by $\mathrm{FER}_{k}(\gamma ),$ and it can be estimated as follows:
    25 $$\begin{aligned} \mathrm{FER}_{k}(\gamma )=\begin{Bmatrix} 1&,if 0<\gamma <\gamma _{n}, \\ 1-(1-P_{e})^{NL}&,if \gamma \geqslant \gamma _{n}, \end{Bmatrix} \end{aligned}$$
  The probability density function (PDF)$p(\gamma )=\frac{1}{\gamma _{0} }exp(-\frac{\gamma }{\gamma _{0} })$ [30] for the $\gamma$ frame can be used to determine the equation, which selects the mode k, as follows:
    $$\begin{aligned} \zeta _{k}=\int _{\Gamma _{k} }^{\Gamma _{k+1}}p(\gamma ) \mathrm{d}\gamma , k=1,\ldots ,K. \end{aligned}$$
    As a result, PDF becomes as follows:
    26 $$\begin{aligned} \zeta _{k}=\left( exp\left( -\frac{\Gamma _{k}}{\gamma _{0} } \right) \right) -\left( exp\left( -\frac{\Gamma _{k+1}}{\gamma _{0} } \right) \right) \end{aligned}$$
  In Eq. (25), no transmission exists if the channel quality below $\gamma _{n} ; \gamma _{n}$ could be specified by solving the following:
    27 $$\begin{aligned} \int _{\gamma _{n} }^{\infty }1-(1-P_{e}(\gamma _{n}) )^{NL} p(\gamma )\mathrm{d}\gamma =1 \end{aligned}$$
  Let $\overline{\mathrm{FER}_{k}}$, for mode k, present the average FER (i.e., the ratio of the number of incorrectly received packets over transmitted packets) and be mathematically represented as follows:
    28 $$\begin{aligned} \overline{\mathrm{FER}_{k}}=\frac{1}{\zeta _{k}}\int _{\Gamma _{k} }^{\Gamma _{k+1}}1-(1-P_{e}(\gamma ) )^{NL} p(\gamma ) \mathrm{d}\gamma \end{aligned}$$
  FER is considered a performance indicator of the link layer in the reward function. The utility function per frame is presented as follows:
  29 $$\begin{aligned} \frac{\alpha *B*\log _{2}(1+\omega *10^{\frac{\gamma _{0} }{10}})*\frac{NL}{R}*S(\gamma ,M)*\log _{2}(M)}{{\overline{\mathrm{FER}}}} \end{aligned}$$

Thus, the reward function is expressed as:

30 $$\begin{aligned} R(\gamma , M)=\begin{Bmatrix} \frac{\alpha *B*\log _{2}(1+\omega *10^{\frac{\gamma _{0} }{10}})* \frac{NL}{R}*S(\gamma ,M)*\log _{2}(M)}{{\overline{\mathrm{FER}}}}\\ ,if \gamma \geqslant \gamma _{n}, \\ \\ 0 \\ ,otherwise \quad or \quad \gamma < \gamma _{n}, \end{Bmatrix} \end{aligned}$$

where ($\gamma$) is a state, and (*M*) is the action that can be taken by the control agent.

#### Optimal dynamic programming solution

Maximizing the objective function through determining the decision policy $\pi : S\rightarrow A$ is involved in the MDP’s solution. Hence, various typical objective functions are introduced such as discounted and average rewards. As the proposed solution in this paper is based on maximizing throughput per minimizing FER, the MDP policy iteration for average cost method is implemented. This method fits the optimal equation Bellman that requires knowledge of the state transition probability [31] for all $s \epsilon S$.

31 $$\begin{aligned} \eta ^{*}+h^{*}(s)=\underset{a\rightarrow A(s)}{max}\left[ R(s,a)+\sum _{{s}'\epsilon 1}^{|S|}P_{s,{s}'}(a)h^{*}({s}') \right] , \end{aligned}$$

where $\eta ^{*}$ is the optimal average reward per stage and $h^{*}(s)$ is called optimal relative state-value function for each state s.

## Formulation

### Finite-State Markov Channel (FSMC) formulation

**Fig. 6:**
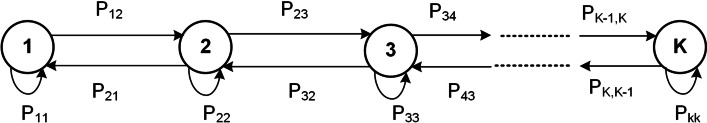
K-state Markov transition channel model [14]

The correlation fading process structure can be described through the effective FSMC approach. FSMC is used to model the dynamic of wireless channel in point-to-point communication [32]. FSMC divides the target SNR ($\Gamma$) into a finite number of intervals (*K* intervals), $0=\Gamma _{0}< \Gamma _{1}<\cdots <\Gamma _{k}$. For every time duration, a channel transition occurs, and SNR is calculated. In addition, the transition of channel occurs between two adjacent states as shown in Fig. 6. On the basis of [32], the transition probability state can be determined as follows:

1. Steady-state probabilities:
  32 $$\begin{aligned} \tau _{k}=\int _{\Gamma _{k} }^{\Gamma _{k+1}}p(\gamma ) \mathrm{d}\gamma , k=1,\ldots ,K. \end{aligned}$$
  $\gamma$ is exponentially distributed with PDF. Hence, similar to Eq. (26)
  $$\begin{aligned} \tau _{k}=\left( exp\left( -\frac{\Gamma _{k}}{\gamma _{0} } \right) \right) -\left( exp\left( -\frac{\Gamma _{k+1}}{\gamma _{0} } \right) \right) \end{aligned}$$
2. State transition probabilities:
  33 $$\begin{aligned} p_{ct}(k,k+1)= & {} \frac{LCF(\Gamma _{k+1})*T_{f}}{\tau _{k}} \quad , k=1,\ldots ,K-1. \end{aligned}$$
  34 $$\begin{aligned} p_{ct}(k,k-1)= & {} \frac{LCF(\Gamma _{k})*T_{f}}{\tau _{k}} \quad , k=2,\ldots ,K. \end{aligned}$$
  where *LCF* is the level crossing function that measures the rapidity of fading.

### Building state transition probabilities

The state transition probability can be built as follows: First, determine the SNR state as $s\equiv (\gamma )$; second, determine the action, which is the modulation level represented as $a\equiv (\textit{M})$; and third, determine the state transition probability, which is denoted by $(\gamma )*(\gamma )*(\textit{M})\rightarrow [0,1]$. Principally, the state transition probability constitutes the channel transition probability and the successful frame transmission probability.

In this respect, the channel is modeled as FSMC, and the $\gamma$ state transition probability is calculated on the basis of the FSMC formulation section and denoted as $P_{ct}(\gamma _{k}, \gamma _{k+1})$. In addition, the successful frame transmission probability $S(\gamma ,\textit{M})$ based on the SNR and modulation is denoted as $S(\gamma ,\textit{M})=(1-P_{e}(\gamma ,\textit{M}))^{NL}$.

Suppose the current state is $S_{k}=(\gamma _{k})$, where $\gamma _{k}$ is SNR and the action chosen at time interval k is $a_{k}=(\textit{M}_{k})$, where $\textit{M}_{k}$ is the modulation level. The state transition probability is specified by considering that the channel transition and successful frame transmission are independent as represented below:

1. Successful transmission:
  35 $$\begin{aligned}&S_{k+1}=(\gamma _{k+1})\nonumber \\&P_{S_{k},S_{k+1}}(a_{k})=S(\gamma ,M_{k}) P_{ct}(\gamma _{k}, \gamma _{k+1}) \end{aligned}$$
2. Failed transmission:
  36 $$\begin{aligned}&S_{k+1}=(\gamma _{k+1})\nonumber \\&P_{S_{k},S_{k+1}}(a_{k})=(1-S(\gamma ,M_{k})) P_{ct}(\gamma _{k}, \gamma _{k+1}) \end{aligned}$$

## Evaluation criteria

The current research evaluated the performance of the existing LTE/LTE-A system, CLLA, and MDP-CLLA in terms of throughput and packet loss for orthogonal frequency-division multiple access (OFDMA) modulation scheme with different packet sizes. In addition, it calculated overhead packet size to determine optimal packet size and then the best phase productivity to determine the best model for each packet size.

### Throughput and packet loss view

Regarding throughput and packet loss, the packet size comprises the MAC header apart from its payload. Therefore, the MAC header is excluded when calculating the throughput and packet loss.

### Overhead packet size (phase productivity) view

This subsection discusses the meaning behind the overhead packet size including the method of determining the number of packets and the meaning of phase productivity that helps in the process of determining overhead packet size and best phase productivity among models.

The overhead discussed in this subsection has two types. The first is header size overhead (space overhead), and the second is modulation switching overhead (processing overhead).

The space overhead is actually the bits that are used for MAC header protocol. In this research, these bits are excluded when the throughput is calculated. In the LTE/LTE-A system, the frame size is fixed on the basis of the modulation type. Its size is approximately 26,400 bits in the case of QPSK. For example, in the case of having 1000 bits of packet size, 27 packets are required to fill the frame bits, whereas only 4 packets are required for 8000 bits packet size. Hence, the overhead of 1000 bits packet becomes higher than that with 8000 bits.

The processing overhead is the overhead that results from the processing time, which results from switching between modulations. Indirectly, SNR distance ($\mathrm{SNR}_{\mathrm{dist}}$), which is referred to the difference between the SNR threshold values of the current modulation and the next, represents the processing delay, which is required when redundant modulation switching overhead crosses SNR threshold.

The overhead can be helpful by determining the adaptive packet size used in the link adaptation. Therefore, in the simulation result section, the overhead is indirectly evaluated and viewed as determining the optimal packet size needed in transmission with a certain modulation. It is called, the phase productivity of packet size at a specific modulation ($\mathrm{phase}_{\mathrm{pktsize}}\mathrm{(modtype)}$). Phase productivity is defined by multiplying the total throughput at specific modulation to the difference value between the SNR threshold values of the current modulation and the next. This phase productivity aims to determine the optimal packet size and the best model at a specific packet size. The ($\mathrm{phase}_{\mathrm{pktsize}}\mathrm{(modtype)}$) is calculated for every packet size and modulation type by:

1. Compute the $\mathrm{SNR}_{\mathrm{dist}}$ as: $\mathrm{SNR}_{\mathrm{dist}}=\mathrm{SNR}_{\mathrm{upper thresh}}-\mathrm{SNR}_{\mathrm{lower thresh}}$.
2. Compute the summation throughput $\sum _{\mathrm{SNR}_{\mathrm{upper thresh}}}^{\mathrm{SNR}_{\mathrm{lower thresh}}} throughput(modtype)$ of the modulation stage to show the total throughput of every modulation stage.
3. Compute the phase productivity of packet size for every modulation stage as: $\mathrm{phase}_{\mathrm{pktsize}} \mathrm{(modtype)}=\mathrm{SNR}_{\mathrm{dist}}*\sum _{\mathrm{SNR}_{\mathrm{upper thresh}}}^{\mathrm{SNR}_{\mathrm{lower thresh}}} {throughput(modtype)}$

**Fig. 7:**
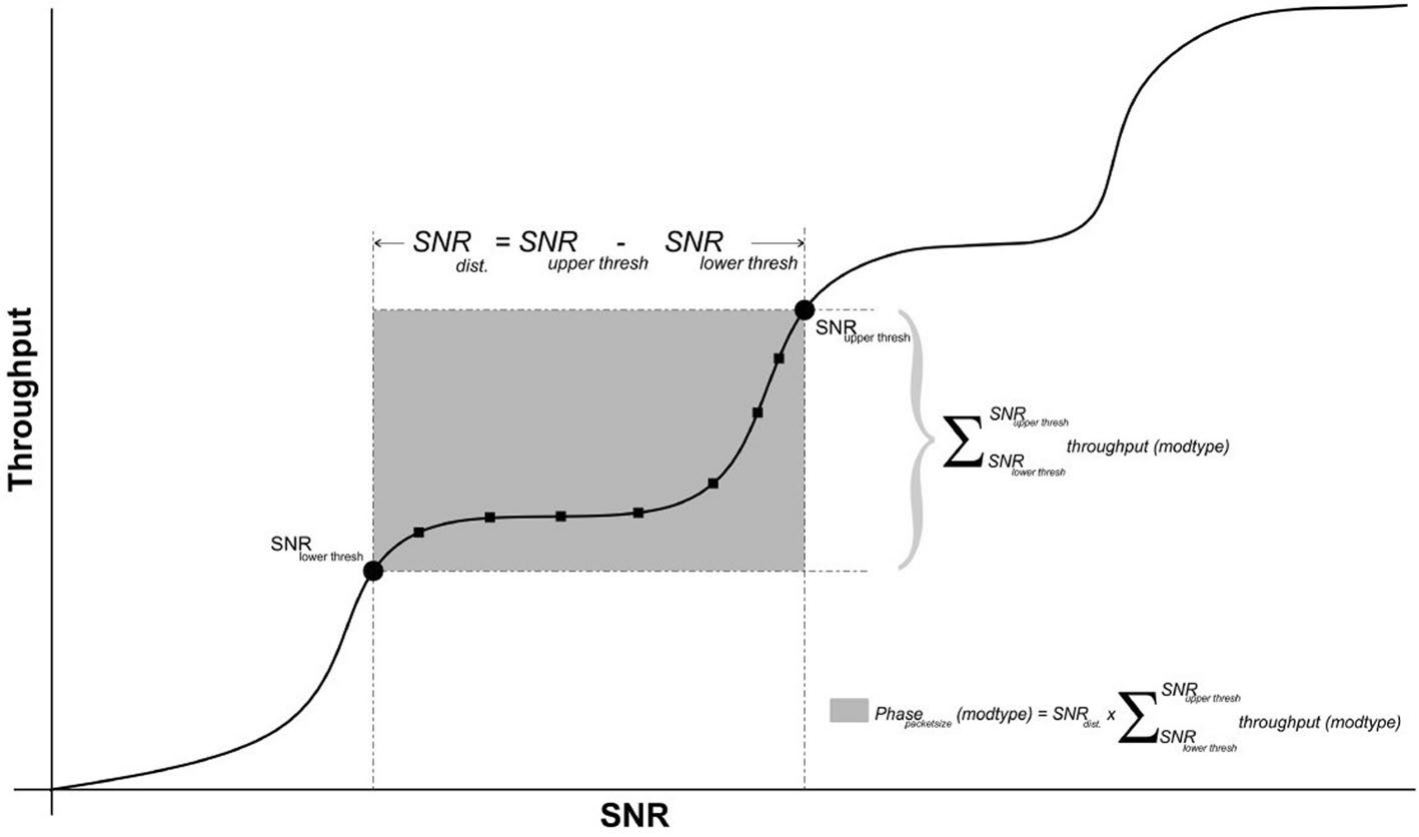
Productivity phase calculation

Then, for every modulation stage, the packet size with the highest productivity value is the optimal packet size because of the following scenarios: Either it is taking care of the stability at that modulation stage, or it has the highest total throughput produced at that modulation stage compared with other packet sizes or both of them. Figure 7 simplifies the phase productivity calculation. For further details, a delivery of data at size of QPSK 1000 bits is considered in the MDP-CLLA model. Then, the period of delivering data    $\mathrm{SNR}_{\mathrm{dist}}=\mathrm{SNR}_{\mathrm{upper thresh}}-\mathrm{SNR}_{\mathrm{lower thresh}} = (42.78 - 35.79)=6.99$ and the summation of throughput on that period is 1611.8 mbps, Hence, the phase productivity in this case is    $6.99 * 1611.8 = 11266.5$. see (Tables 6 and 11).

## Simulation environment

The simulation of LTE consists of a base station (eNodeB) and a UE, where data are transferred from eNode B to UE. The transmission mode 4 (closed loop) is implemented on the basis of 3GPP TS 36.211 V15.6.0 (2019-06) standard release. A channel knowledge at the transmitter is required at closed loop and occurs when dynamic adjustment is executed on the basis of the feedback of UEs. In addition, PTP is the type of connection between eNodeB and UE. Table 3 illustrates the summarized parameters of the LTE simulation.

**Table 3:** Parameters setting of LTE simulation

Parameters	Setting
Carrier Frequency	1.993 GHz
Sub-carrier Spacing	15 kHz
Transmission Bandwidth	20 MHz
Transmission Mode	Closed loop (TM4)
Channel Model	Rayleigh
Modulation Type	QPSK, 16-QAM, 64-QAM
MAC Header Type and Size	R/R/E/LCID/F/L (2 Byte)

## Simulation result

The results of the existing LTE system, the CLLA model, and the MDP-CLLA model on the bases of throughput and packet loss are discussed here. Consequently, according to [33–36], the transmission video data in this simulation are encoded with an H264 video coder at packet sizes of 125; 250; 500; and 1000 bytes, which reflect to 1000; 2000; 4000; and 8000 bits, respectively. The simulation was run 20 times, and each run includes 6000 frames. The average throughput and packet loss results are analyzed on the bases of SISO mode and on various packet sizes (see Table 1) over Rayleigh fading channel by considering that the packet size comprises, in addition to its payload, the MAC header. Consequently, the MAC header is excluded to the payload when calculating the throughput.

### Throughput results

This subsection describes the throughput results in the case of SISO mode with various packet sizes. In this respect, the corresponding data rates of SNR threshold values for various packet size are presented. The purpose of these experiments is to show the effect of increasing packet size at different modulation levels to the throughput result on the proposed models compared with the existing system.

**Fig. 8:**
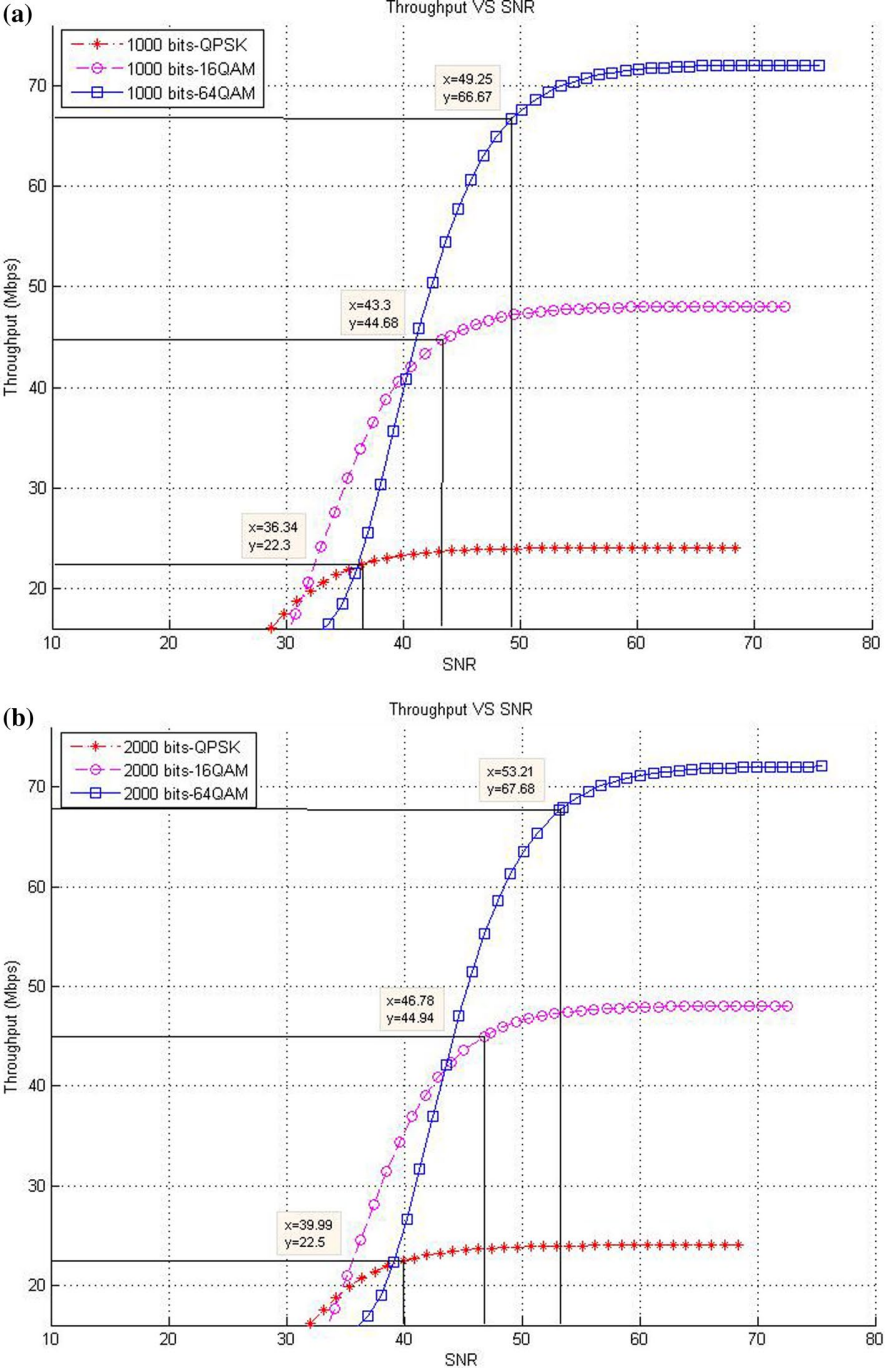

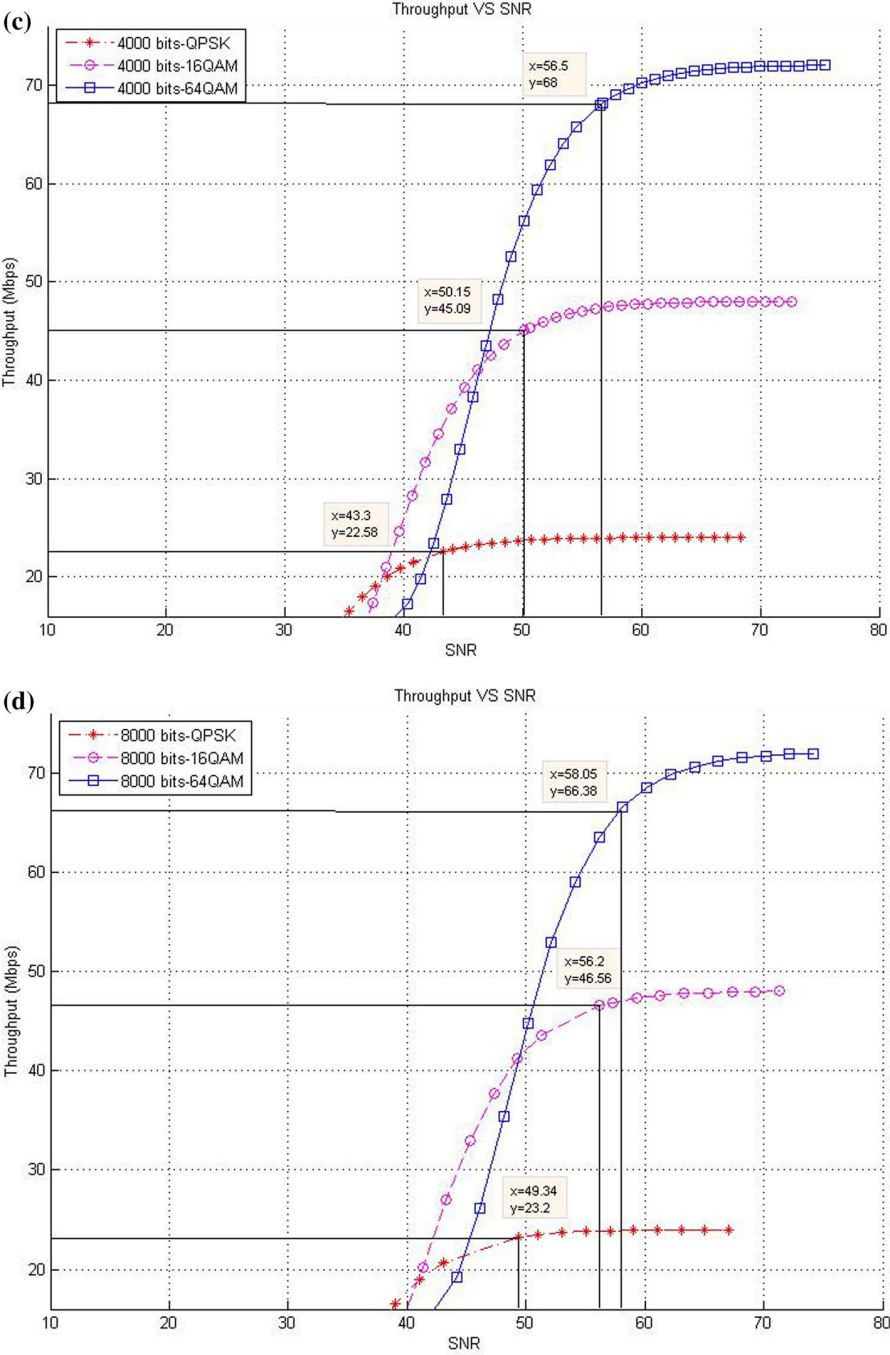
**a** 1000 bits, **b** 2000 bits, **c** 4000 bits, **d** 8000 bits (**a**–**d**) throughput versus SNR for various packet sizes and modulation in the existing LTE system [13]

**Fig. 9:**
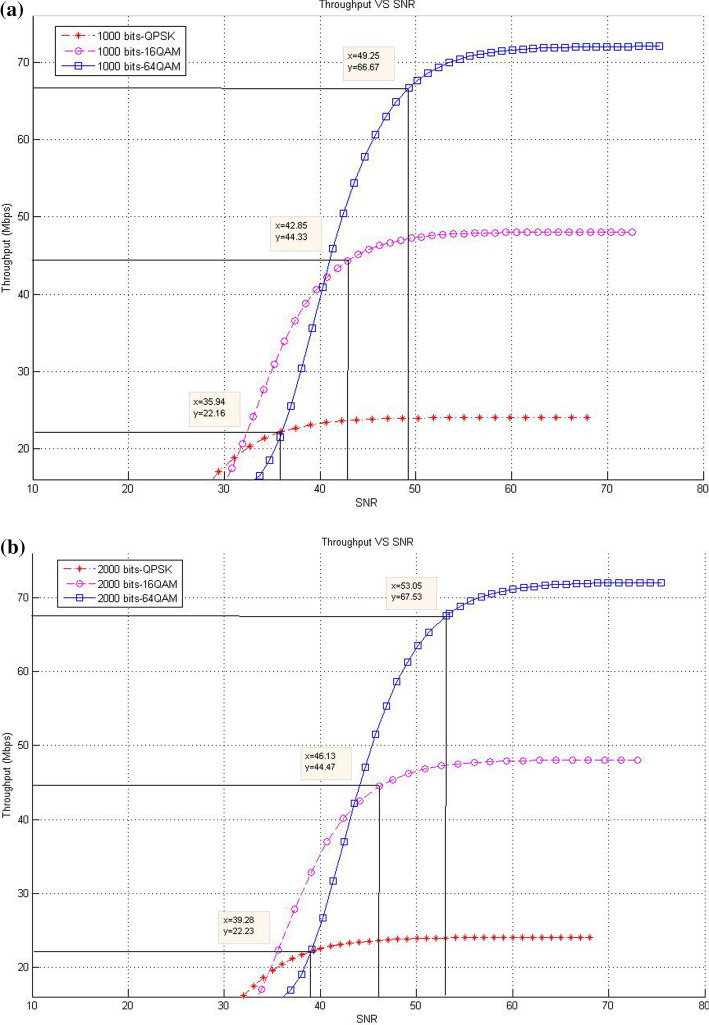

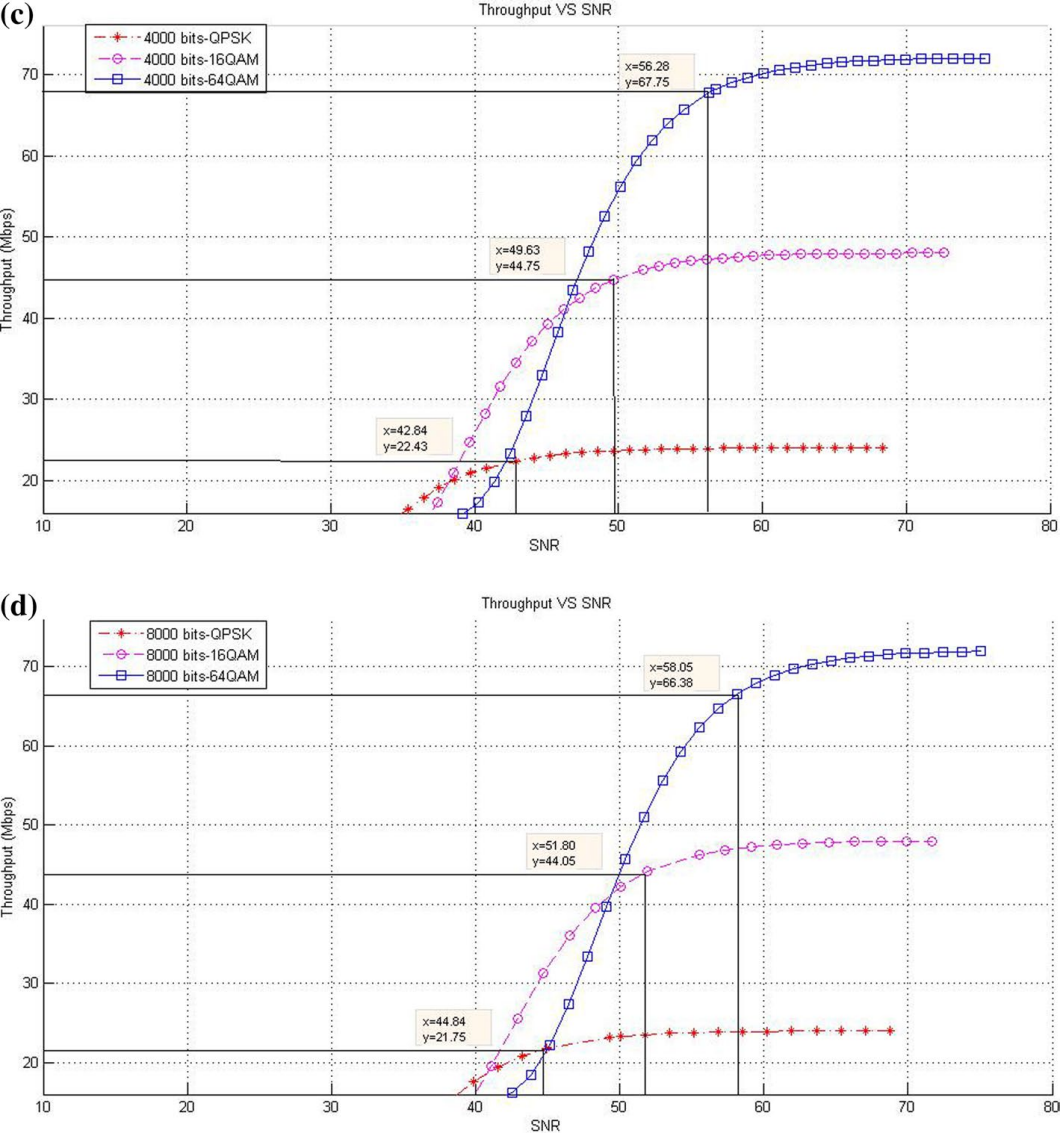
**a** 1000 bits, **b** 2000 bits, **c** 4000 bits, **d** 8000 bits (**a**–**d**) throughput versus SNR for various packet sizes and modulation in the CLLA model

**Fig. 10:**
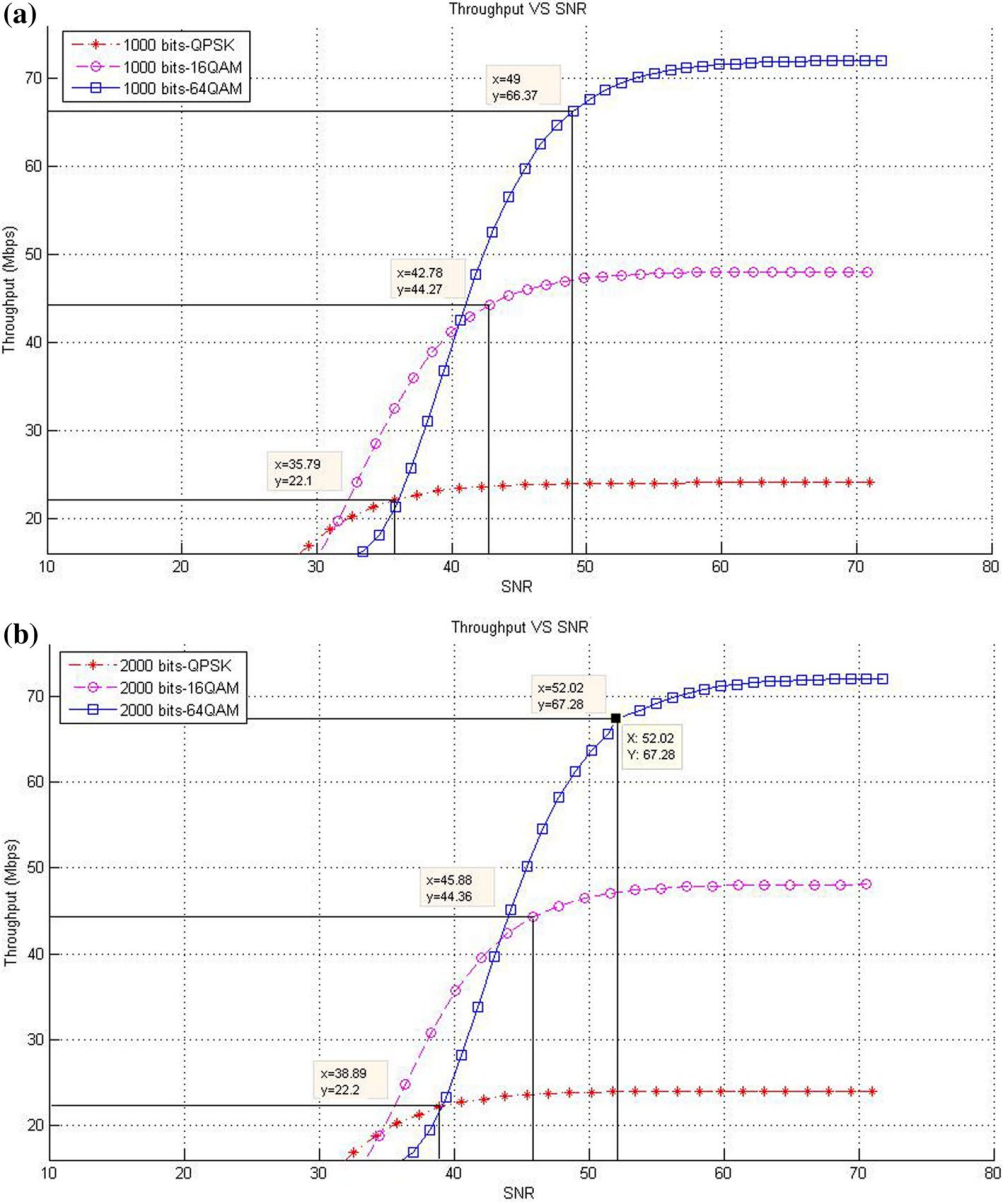

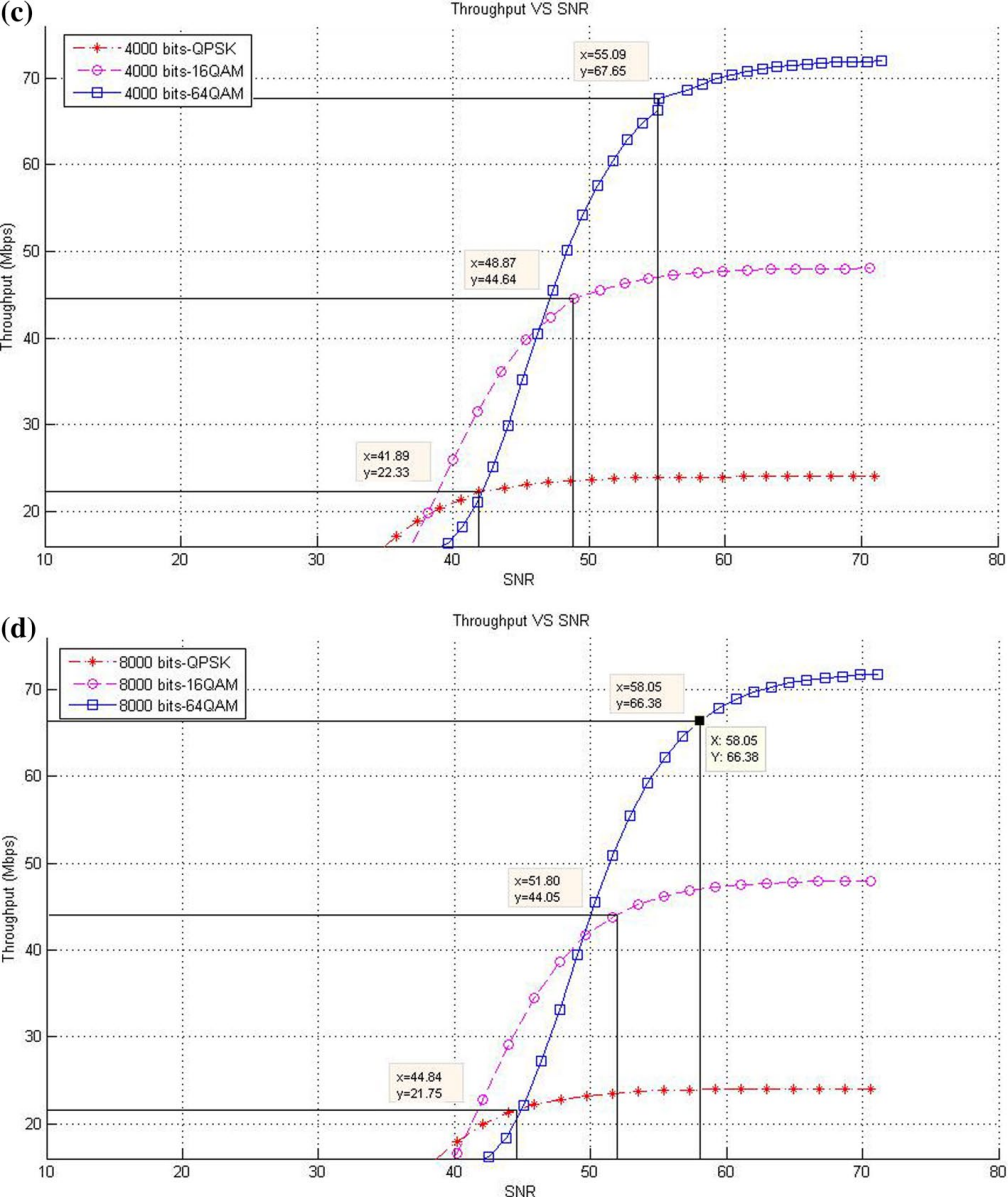
**a** 1000 bits, **b** 2000 bits, **c** 4000 bits, **d** 8000 bits (**a**–**d**) throughput versus SNR for various packet sizes and modulation in the MDP-CLLA model

- *Existing LTE system*
  In the existing LTE system, Fig. 8a–d illustrates the throughput measurements for different sizes of packet (see Table 1), with the related SNR threshold values needed to deliver the data. To illustrate, for the packet size of 1000 bits, Fig. 8a shows that the SNR threshold values (36.34, 43.3, and 49.25 dB) are corresponding to the throughput values (22.3, 44.68, and 66.67 Mbps) for QPSK, 16-QAM, and 64-QAM, respectively. The summary results of Fig. 8a–d are illustrated in Table 4.
  As shown in Fig. 8, the corresponding throughput of SNR threshold values is increased when the packet size is increased, except for the case when it reaches 8000 bits and the modulation is 64-QAM. This case resulted in a slight decrease in the throughput than that in other packet sizes of 1000; 2000; and 4000 bits because packet size has a negative effect on the throughput at a certain level. This finding can be justified as follows: For a big packet size, the packet loss has a considerable impact on the throughput due to the amount of data lost as the network with big packet size and higher SNR would not be able to handle such load. Therefore, noticing a lower throughput is expected when the packet size reaches to a certain level (e.g., 8000 bits).
  Furthermore, increasing packet size leads to a decrease in the number of packets for each frame, thereby decreasing the total MAC header data. At the end, the peak data rate is increased because of the less total MAC header data excluded from throughput computation.
- *CLLA Model*
  In the CLLA model, Fig. 9 illustrates the same measurement factors as in (8).
  As shown in Fig. 9, if the packet size is increased, then the corresponding throughput of the SNR threshold values is also increased, unless the packet size reaches 8000 bits. This exceptional case is decreased compared with 1000; 2000; and 4000 bits as packet size, where its throughput values at QPSK, 16-QAM, and 64-QAM are 21.75, 44.05, and 66.38, respectively. It is observed that the packet size has negative impact on the throughput at 8000 bits packet size because of the traffic load handling. Table 5 highlights the SNR threshold values with their corresponding throughput for various packet sizes and in different modulation types.
- *MDP-CLLA Model*
  In the MDP-CLLA model, Fig. 10 illustrates the similar behavior results as in the CLLA model, where the corresponding throughput of the SNR threshold values is also increased in case the packet size is increased. However, an exceptional case occurs from the above results when the packet size is 8000 bits. In this case, the throughput values are decreased compared with the other packet sizes. As an example, the throughput values at 1000; 2000; 4000; and 8000 bits of QPSK modulation are 22.1, 22.2, 22.33, and 21.75 Mbps, respectively. The packet size of 8000 bits apparently detects PER at the accepted 10% or close to it and has a negative effect on the throughput than that of other packet sizes because of load handling. Hence, it has a direct effect on the throughput. Table 6 highlights the SNR threshold values with their corresponding throughput in the MDP-CLLA model.

**Table 4:** SNR and corresponding throughput values for various packet sizes in the existing LTE system

Packet size	Modulation type					
	QPSK		16-QAM		64-QAM	
	SNR (dB)	Throughput (Mbps)	SNR (dB)	Throughput (Mbps)	SNR (dB)	Throughput (Mbps)
1000	36.34	22.3	43.3	44.68	49.25	66.67
2000	39.99	22.5	46.78	44.94	53.21	67.68
4000	43.3	22.58	50.15	45.09	56.5	68
8000	49.34	23.2	56.2	46.56	58.05	66.38

**Table 5:** SNR and corresponding throughput values for various packet sizes in the CLLA model

Packet size	Modulation type					
	QPSK		16-QAM		64-QAM	
	SNR (dB)	Throughput (Mbps)	SNR (dB)	Throughput (Mbps)	SNR (dB)	Throughput (Mbps)
1000	35.94	22.16	42.85	44.33	49.25	66.67
2000	39.28	22.23	46.13	44.47	53.05	67.53
4000	42.84	22.43	49.63	44.75	56.28	67.75
8000	44.84	21.75	51.8	44.05	58.05	66.38

**Table 6:** SNR and corresponding throughput values for various packet sizes in the MDP-CLLA model

Packet size	Modulation type					
	QPSK		16-QAM		64-QAM	
	SNR (dB)	Throughput (Mbps)	SNR (dB)	Throughput (Mbps)	SNR (dB)	Throughput (Mbps)
1000	35.79	22.1	42.78	44.27	49	66.37
2000	38.89	22.2	45.88	44.36	52.02	67.28
4000	41.89	22.33	48.87	44.64	55.09	67.65
8000	44.84	21.75	51.8	44.05	58.05	66.38

### Packet loss results

This part presents a scenario of the accepted packet loss for a given SNR threshold values in various packet sizes. This scenario includes three different experiments on the bases of the modulation type where the results are presented in terms of different packet sizes (see Table 1).

**Fig. 11:**
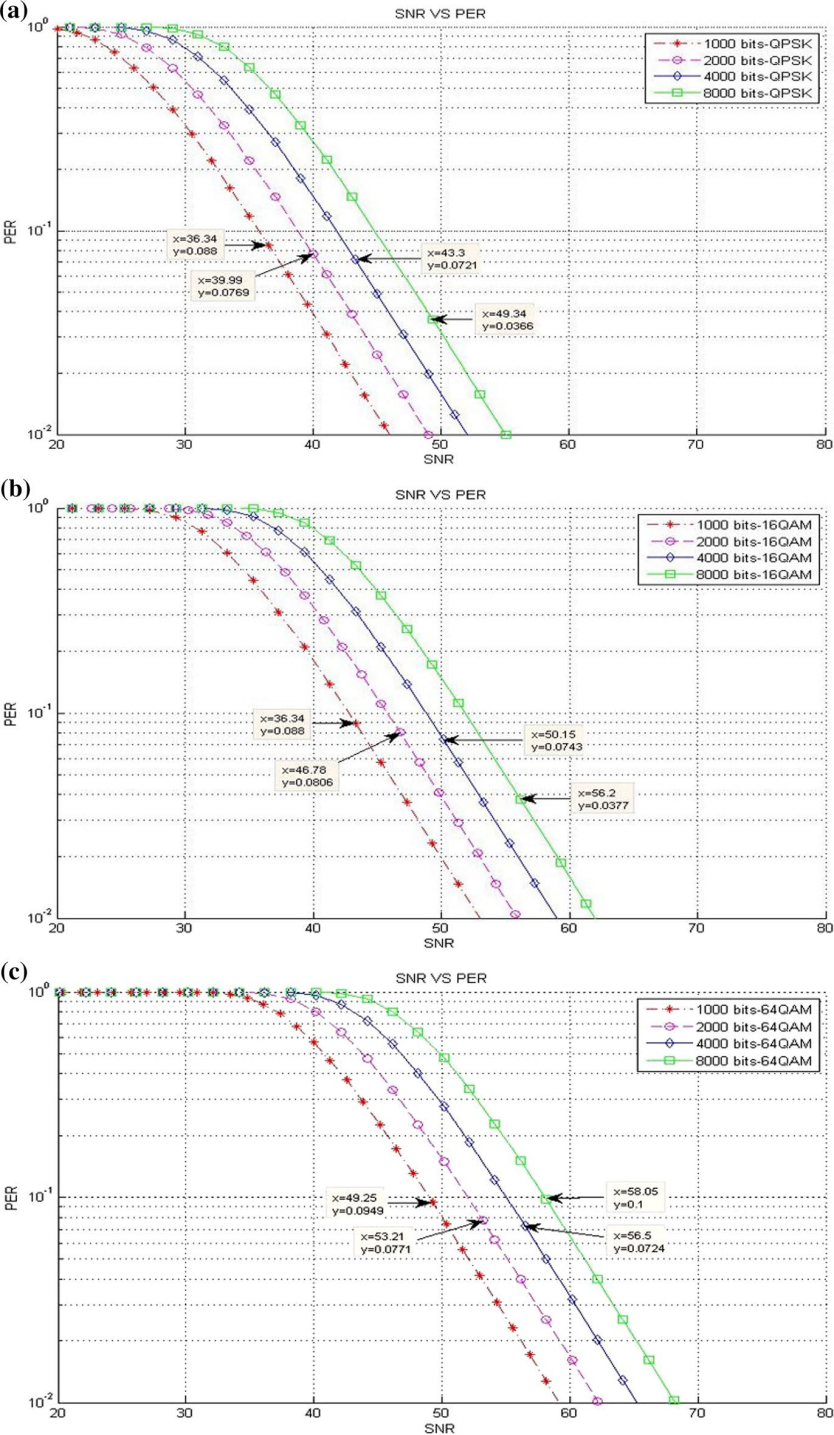
**a** QPSK, **b** 16-QAM, **c** 64-QAM (**a**–**c** packet loss of SNR threshold values for different modulation types and various packet sizes in the existing LTE system [13]

**Fig. 12:**
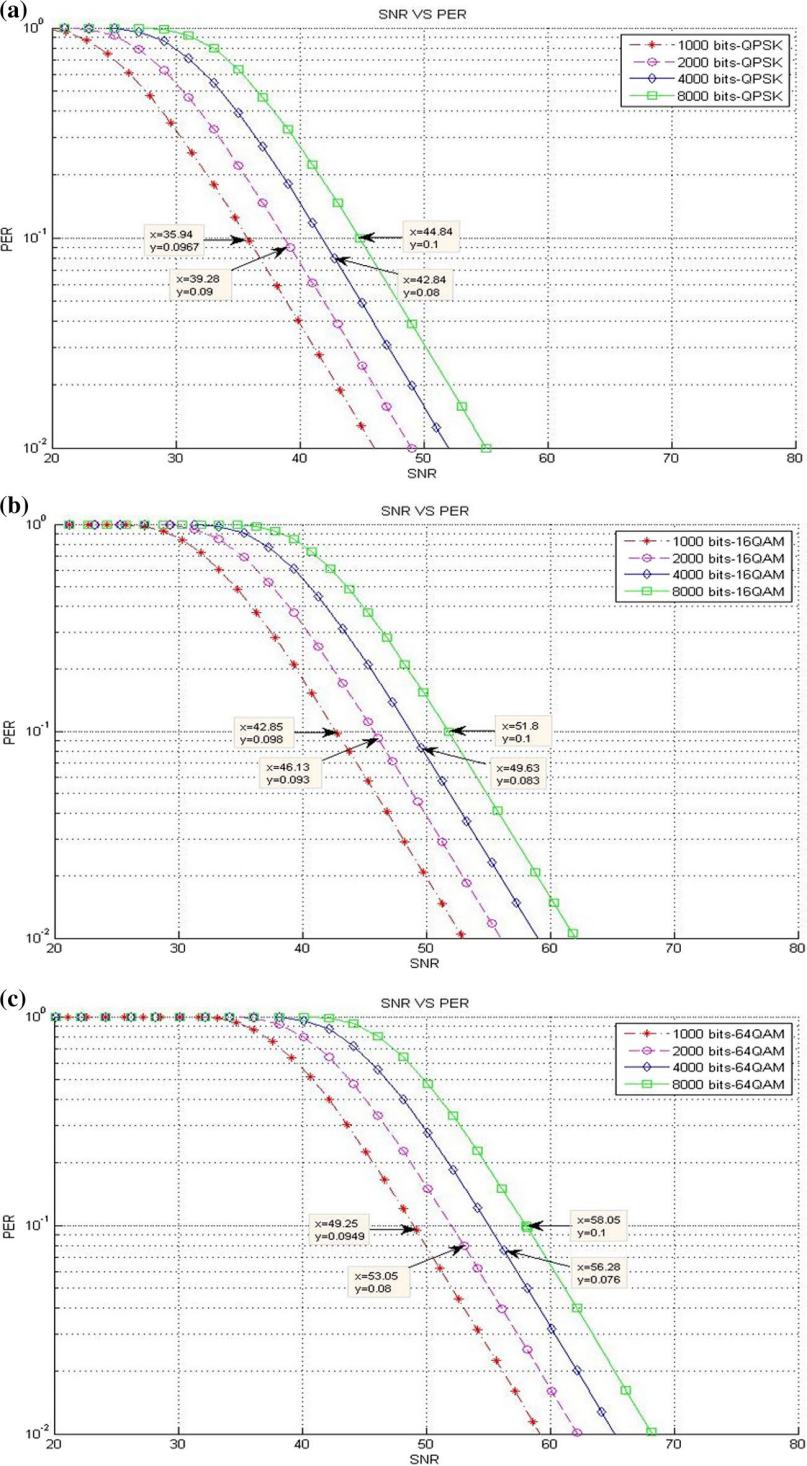
**a** QPSK, **b** 16-QAM, **c** 64-QAM (**a**–**c**) packet loss of SNR threshold values for different modulation types and various packet sizes in the CLLA model

**Fig. 13:**
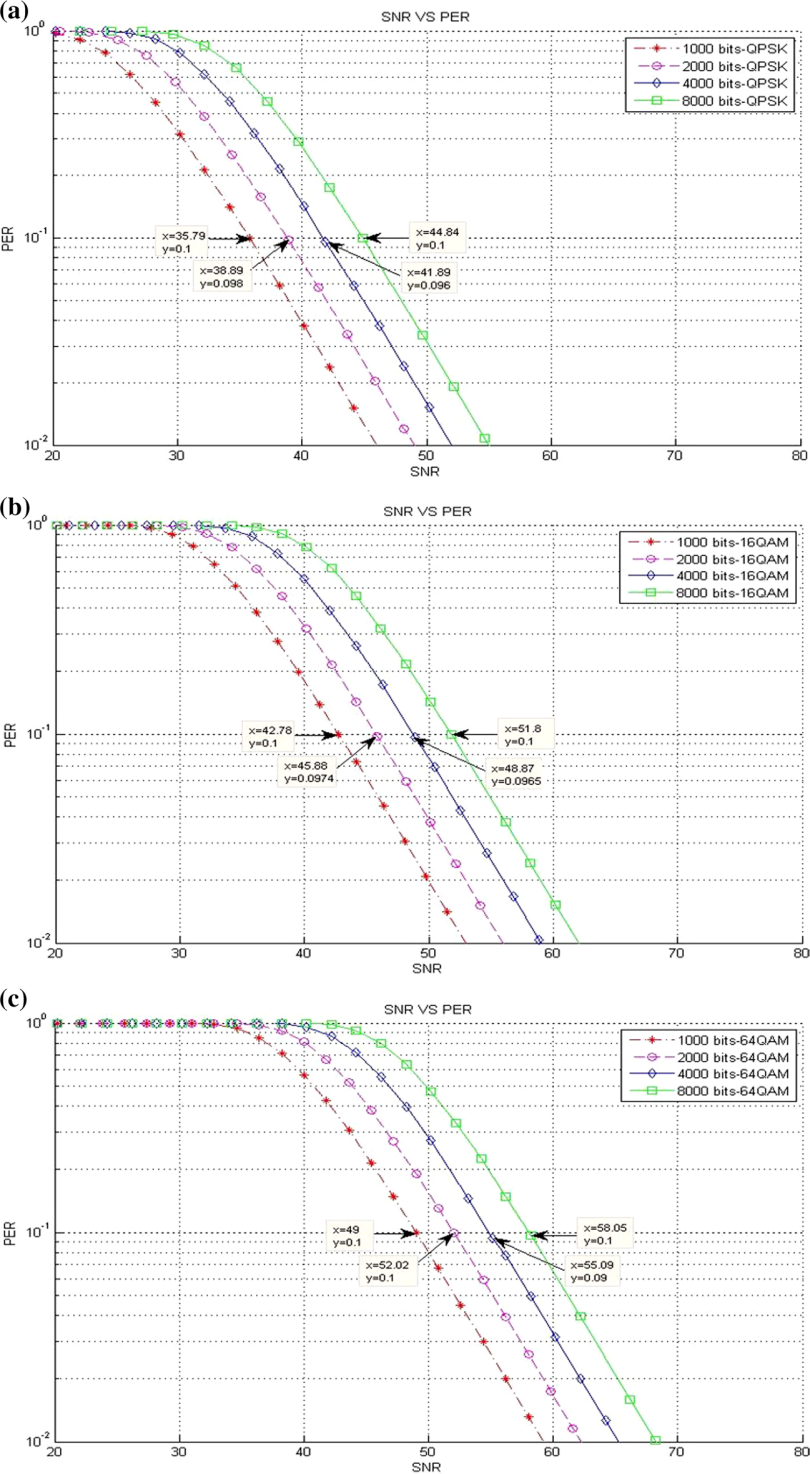
**a** QPSK, **b** 16-QAM, **c** 64-QAM (**a**–**c**) packet loss of SNR threshold values for different modulation types and various packet sizes in the MDP-CLLA model

- *Existing LTE system*
  Figure 11 clarifies the packet loss at different SNR threshold values in the existing LTE system. Clearly, a reduction happens in the packet loss when the packet size is increased. This finding is true for all packet sizes, unless the packet size is at 8000 bits, while the modulation is 64-QAM. This result is due to the sensitivity of packet size to packet loss, and this behavior causes the packet loss to drop down rapidly from an unacceptable level at ($> \approx 10\%$) to acceptable level at ($<< 10\%$).
- *CLLA Model*
  Figure 12 illustrates the packet loss at different SNR threshold values in the CLLA model. Obviously, the packet loss is reducing when the packet size is increased; the packet loss tendency differs only for the packet size of 8000 bits because of its sensitivity to packet loss.
- *MDP-CLLA Model*
  As shown in Fig. 13, the packet loss versus SNR is illustrated where the packet loss is decreased when packet size is increased for 1000; 2000; and 4000 bits of packet size. Conversely, an increase happens in the packet loss in the case of 8000 bits packet size than those of 1000; 2000; and 4000 bits packet size. The reduction in packet loss at the corresponding SNR threshold values occurs slightly and is close to or reaches 10%. This finding implies that the MDP-CLLA model is more accurate for perceiving the adaptation level than the CLLA model and the existing system. Hence, the switching from one modulation type to another is more accurate with the MDP-CLLA model than the other models.

- ***Discussion of Comparison among the Models based on throughput and packet loss***
  Switching from modulation to another is presented by determining the adaptive switching SNR range (ASR) which includes the range among the SNR switching points of the MDP-CLLA model, the CLLA model, and the existing LTE system. The purpose of this switching level is to compare the improvement of throughput among different models. Switching modulation can be (QPSK to 16-QAM) and (16-QAM to 64-QAM). As shown in Figs. 11b, 12b, and 13b, for example, when the packet size stands at the point of 2000 bits, ASR for switching from QPSK to 16-QAM is [45.88-46.78]. In this switching modulation level, the PERs are 0.0806, 0.093, and 0.0974 which correspond to 46.78, 46.13, and 45.88 dB as SNR threshold values in the existing system, the CLLA model, and the MDP-CLLA model, respectively. This finding indicates that the link adaptation at the CLLA model achieved noticeable enhancement in throughput than the existing LTE system. In addition, the link adaptation in the MDP-CLLA model achieved best enhancement in throughput over the existing LTE system and the CLLA model.
  To elaborate this finding, the comparison between the CLLA model and the existing system (CLLA$\_$Existing system) is considered. Then, at 46.5 dB, the link adaptation at the CLLA model is switched from QPSK to 16-QAM, whereas the existing system remains at QPSK. Consequently, the throughput value of the CLLA model is 44.76 Mbps, while it is 23.68 Mbps for the existing system. In addition, the comparison among MDP-CLLA, CLLA, and the existing system (MDP-CLLA$\_$CLLA$\_$Existing system) is considered. Then, the throughput measurement at 46 dB is 44.42 Mbps for the MDP-CLLA model, while it is 23.64 Mbps for the CLLA model and the existing system as well. This finding is explained by the fact that the MDP-CLLA model has switched to 16-QAM, whereas the CLLA model and the existing system remain at QPSK.
  From the above example, the CLLA model is improved by 89% than the existing LTE system at 46.5 dB. Furthermore, the MDP-CLLA model is improved by 87.9% than the CLLA and the existing system at 46 dB. The same behavior also results when switching from 16-QAM to 64-QAM.
  Table 7 shows the throughput results with different packet sizes in (CLLA$\_$Existing system) and the improvements of the CLLA model when compared with the existing system. In addition, Table 8 shows the throughput results with different packet sizes in (MDP-CLLA$\_$CLLA$\_$Existing system) and the improvements of the MDP-CLLA model compared with the CLLA model and the existing system. Generally, the results have shown that the throughput in the CLLA model is improved by 87.5–89.6% and by 0–43.3% for (QPSK $\rightarrow$ 16-QAM) and (16-QAM $\rightarrow$ 64-QAM) switching modulation, respectively, compared with the existing system. Moreover, the throughput of the MDP-CLLA model is improved by 87.5–88.6% and by 0–43.2% for (QPSK $\rightarrow$ 16-QAM) and (16-QAM $\rightarrow$ 64-QAM) switching modulation, respectively, compared with the CLLA model and the existing system.

**Table 7:** Experimental results of CLLA$\_$Existing system

CLLA_Existing system scenario								
Packet size	QPSK $\rightarrow$ 16-QAM				16-QAM $\rightarrow$ 64-QAM			
	SNR point (dB)	Throughput CLLA	Throughput existing system	CLLA enhancement (%)	SNR point (dB)	Throughput CLLA	Throughput existing system	CLLA enhancement (%)
1000	43	44.45	23.65	87.9	49.25	66.67	66.67	0
2000	46.5	44.67	23.68	89	53.1	67.58	47.32	42.8
4000	50	44.99	23.72	89.6	56.4	67.85	47.34	43.3
8000	52	44.22	23.58	87.5	58.05	66.38	66.38	0

**Table 8:** Experimental results of MDP-CLLA$\_$CLLA$\_$Existing system

CLLA_Existing system scenario										
Packet size	QPSK $\rightarrow$ 16-QAM					16-QAM $\rightarrow$ 64-QAM				
	SNR point (dB)	Throughput MDP-CLLA	Throughput CLLA	Throughput existing system	MDP-CLLA enhancement (%)	SNR point (dB)	Throughput MDP-CLLA	Throughput CLLA	Throughput existing system	MDP-CLLA enhancement (%)
1000	42.8	44.33	23.63	23.63	87.6	49.15	66.55	47.15	47.15	41.12
2000	46	44.42	23.64	23.64	87.9	52.5	67.33	47.21	47.21	42.6
4000	49.5	44.65	23.67	23.67	88.6%	55.5	67.67	47.25	47.25	43.2
8000	52	44.22	44.22	23.58	87.5, both MDP-CLLA and CLLA models	58.05	66.38	66.38	66.38	0

### Results and discussion of overhead packet size (phase productivity)

This subsection describes the optimal packet size evaluation for each modulation among models by measuring the phase productivity. Accordingly, the phase productivity for different packet sizes and modulations is presented. The purpose of this result is to determine the optimal packet size and then to obtain the best model at a specific packet size, among the others.

**Fig. 14:**
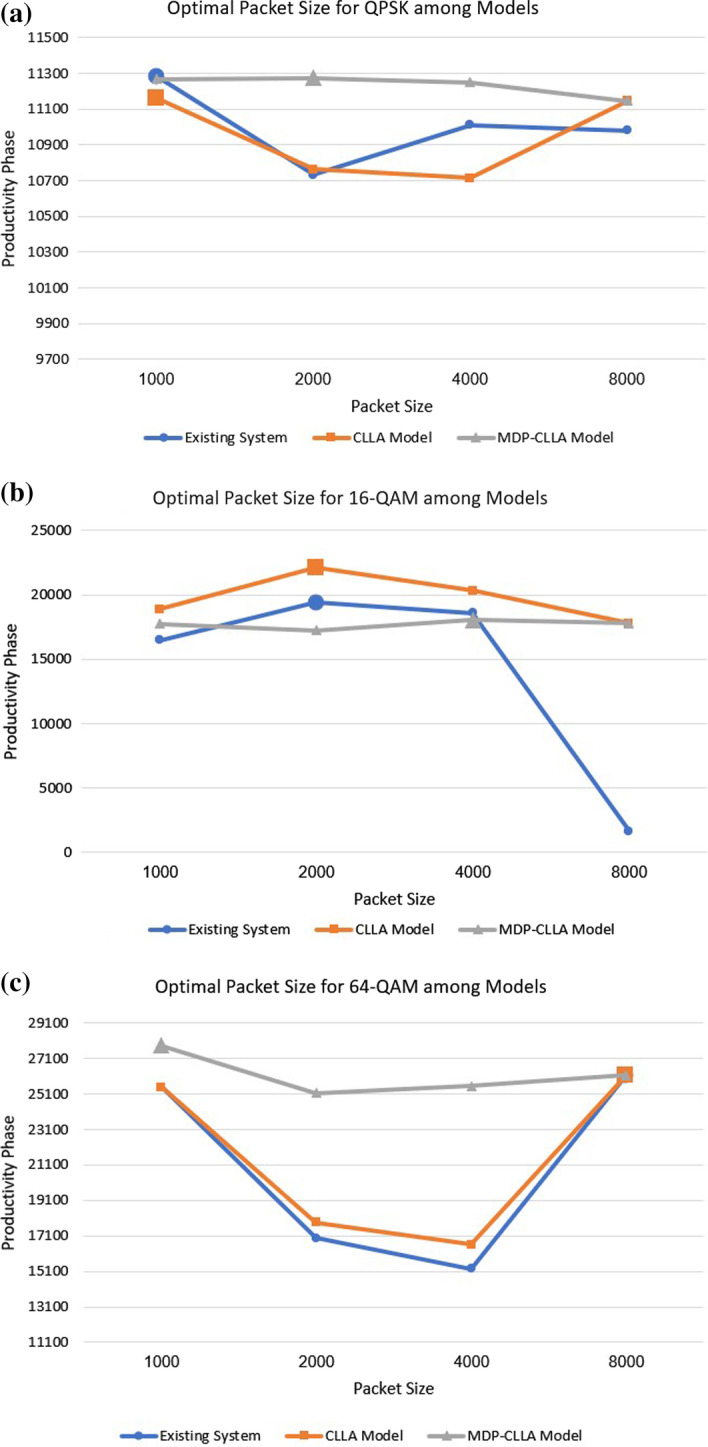
**a** QPSK, **b** 16-QAM **c**, 64-QAM (**a**–**c**) optimal packet size evaluation for each modulation among models

Results presented in Tables 9, 10 and 11 show that for the QPSK, the packet sizes of 1000; 1000; and 2000 bits are considered the optimal packet sizes to be adapted in the existing system, the CLLA, and the MDP-CLLA models, respectively. In the case of 16-QAM, the 2000; 2000; and 4000 bits are the optimal packet sizes in the existing system, the CLLA, and the MDP-CLLA models, respectively. Apparently, the MDP-CLLA model has a larger optimal packet size compared with the other models as shown in Fig. 14, which improves the throughput and channel efficiency. By contrast, for 64-QAM, the 8000; 8000; and 1000 bits are the optimal packet sizes in the existing system, the CLLA, and the MDP-CLLA models, respectively. This exceptional case is due to the SNR distance and the throughput that have a significant impact on the phase productivity results of both existing system and the CLLA model in the case of 8000 bits compared with the other packet sizes. As a conclusion, the MDP-CLLA model has advantage over the existing system and the CLLA model for marginal and moderate signal quality because the optimal packet size is comparatively larger. For 64-QAM which is when signal quality is excellent, performing any dynamic adaptation regarding packet size is unnecessary. The bold values in the phase productivity columns in Tables 9, 10 and 11 reflect the values that correlate to the ideal packet sizes that must be selected.

**Table 9:** Phase productivity for different packet sizes and modulations in the existing system

Packet size	QPSK			16-QAM			64-QAM		
	$\mathrm{SNR}_{\mathrm{dist}}$	$\sum thr$	Phase productivity	$\mathrm{SNR}_{\mathrm{dist}}$	$\sum thr$	Phase productivity	$\mathrm{SNR}_{\mathrm{dist}}$	$\sum thr$	Phase productivity
1000	6.96	1621	**11282**.**1**	5.95	2770.7	16485.7	6.05	4214.6	25498.3
2000	6.79	1580.4	10730.9	6.43	3017	**19399**.**3**	4.89	3468.9	16963
4000	6.85	1607.5	11011.4	6.35	2927.6	18590.3	4.66	3268.2	15229.8
8000	6.86	1600.8	10981.5	1.85	889.7	1646	6.12	4275.5	**26166**.**1**

**Table 10:** Phase productivity for different packet sizes and modulations in the proposed CLLA model

Packet size	QPSK			16-QAM			64-QAM		
	$\mathrm{SNR}_{\mathrm{dist}}$	$\sum thr$	Phase productivity	$\mathrm{SNR}_{\mathrm{dist}}$	$\sum thr$	Phase productivity	$\mathrm{SNR}_{\mathrm{dist}}$	$\sum thr$	Phase productivity
1000	6.91	1615.2	**11161**	6.4	2948.6	18871	6.05	4214.6	25498.3
2000	6.85	1571.2	10762.7	6.92	3190.7	**22079**.**6**	5.05	3536.4	17858.8
4000	6.79	1577.8	10713.3	6.65	3057.5	20332.4	4.88	3430.9	16611
8000	6.96	1601.4	11145.7	6.25	2845.4	17783.8	6.12	4275.5	**26166**.**1**

**Table 11:** Phase productivity for different packet sizes and modulations in the proposed MDP-CLLA model

Packet size	QPSK			16-QAM			64-QAM		
	$\mathrm{SNR}_{\mathrm{dist}}$	$\sum thr$	Phase productivity	$\mathrm{SNR}_{\mathrm{dist}}$	$\sum thr$	Phase productivity	$\mathrm{SNR}_{\mathrm{dist}}$	$\sum thr$	Phase productivity
1000	6.99	1611.8	11266.5	6.22	2853.5	17748.8	6.3	4416.2	**27822**.**1**
2000	6.99	1613	**11274**.**9**	6.14	2806.5	17232	6.08	4137.4	25155.4
4000	6.98	1611.7	11249.7	6.23	2892.8	**18022**.**1**	6.07	4207.6	25540.1
8000	6.96	1601.4	11145.7	6.25	2845.4	17783.8	6.12	4275.5	26166.1

**Fig. 15:**
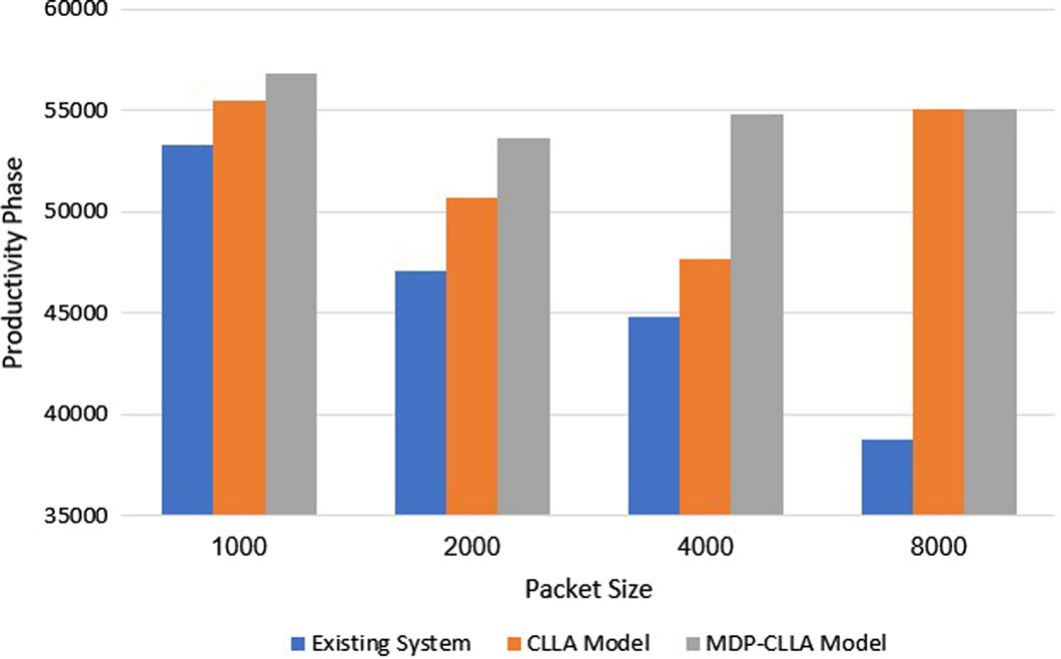
Phase productivity of models for different packet sizes

Regarding the best phase productivity among models, its calculation is performed through the summation of phase productivity for different modulations for each model with each packet size. In the end, for each packet size, the model with the higher phase productivity is the best. Figure 15 shows that the CLLA model improves the phase productivity by 4.25%, 7.66%, 6.3%, and 42.02% for 1000; 2000; 4000; and 8000 bits compared with the existing system. In addition, the MDP-CLLA achieves best improvement in phase productivity by 6.7%, 13.95%, 22.26%, and 42.02% for 1000; 2000; 4000; and 8000 bits compared with the existing system and by 2.35%, 5.84%, 15.01%, for 1000; 2000; and 4000 bits compared with the CLLA model, respectively. By contrast, for 8000 bits, no improvement occurs in the MDP-CLLA model compared with the CLLA model because they both have the same phase productivity.

## Result validation

The validation of results is done by first calculating the summation of the phase productivity for every modulation at a specific packet size. Second, these total results are validated through a statistical method to determine which model has a significant impact. This process is done for the MDP-CLLA model, the CLLA model, and for the existing system.

Validation of results is tested statistically using the Statistical Package for the Social Sciences (SPSS) software. The results were presented as mean ± standard deviation (SD). Statistical analysis of the data was carried out by using one-way analysis of variance (ANOVA) followed by post hoc test. The differences between the means were considered significant at *P* = 0.05, where *P* is defined as the level of statistical significance or the probability of observing the given value of the test statistic, or greater under the null hypothesis. Hence, if *P*< 0.05, then it indicates that the alternative hypothesis has strong evidence against the null hypothesis and vice versa.

For Rayleigh channel, Table 9 illustrates that at 1000; 2000; and 4000 bits, the CLLA and MDP-CLLA models are highly significant than the existing system at positive correlation (P<0.05). In addition, the MDP-CLLA model increased significantly compared with the CLLA model with (P<0.05) at the same packet sizes. In the case of 8000 bits, the CLLA and the MDP-CLLA models are highly significant compared with the existing system with (P<0.05), whereas no obvious increase happens between the MDP-CLLA and the CLLA models in this packet size (P>0.05) because their SNR threshold values are the same.

Obviously, the MDP-CLLA model is proven to be the best efficient model compared with the CLLA and the existing system, as it has the highest significance. Table 12 represents the average total phase productivity of the existing system, the CLLA model, and the MDP-CLLA model for different packet sizes as mean ± SD.

**Table 12:** Phase productivity results as mean SD

Packet size (bits)	1000	2000	4000	8000
Existing system	53266.1 ± 10.1	47093.2 ± 10.2	44831.5 ± 15.3	38793.6 ± 11.7
CLLA model	55530.3 ± 14.8	50701.1 ± 10.9	47656.7 ± 11.7	55095.6 ± 13.1
MDP-CLLA model	56837.4 ± 12.5	53662.3 ± 8.7	54811.9 ± 12.2	55095.6 ± 12.2
$\rho$ value	>**0.05**	<**0.05**	<**0.05**	<**0.05** when comparing CLLA to existing system and MDP-CLLA to existing system. >**0.05** when comparing MDP-CLLA to CLLA

Overall, the outcomes of the MDP-CLLA model become the best in terms of accuracy of link adaptation compared with the CLLA model and the existing system since it is able to find all potential solutions and can identify the optimum utilizing the average reward function.

## Conclusion

This research is aimed to enhance the channel efficiency and throughput within an acceptable error rate by proposing a framework at physical and MAC layers containing two LA models: the CLLA and the MDP-CLLA models. The CLLA model is based on downward cross-layer utilizing analytical prediction of packet loss for the next modulation at the MAC layer beside deterministic SNR threshold values at physical layer. The MDP-CLLA model is proposed over the CLLA model that selects the optimal modulation level more accurately to be used for the next frame transmission. It offers more enhancement in throughput and channel efficiency compared with the CLLA model and the existing LTE system. Generally, the experimental results showed that the throughput in the CLLA model is improved by 87.5–89.6% and by 0–43.3% for (QPSK $\rightarrow$ 16-QAM) and (16-QAM $\rightarrow$ 64-QAM) switching modulation, respectively, compared with the existing system. Moreover, the throughput of the MDP-CLLA model is improved by 87.5–88.6% and by 0–43.2% for (QPSK $\rightarrow$ 16-QAM) and (16-QAM $\rightarrow$ 64-QAM) switching modulation, respectively, compared with the CLLA model and the existing system. Regarding packet sizes, results indicate that at 1000; 2000; and 4000 bits, the LA at the CLLA and the MDP-CLLA models achieved noticeable enhancement in throughput compared with the existing LTE system. In addition, the LA at the MDP-CLLA model increased significantly compared with the CLLA model at the same packet sizes. In the case of 8000 bits, the LA at the CLLA and MDP-CLLA models achieved noticeable enhancement compared with the existing LTE system, whereas no obvious increase occurred between the MDP-CLLA and CLLA models in this packet size because their SNR threshold values are the same. Regarding the best phase productivity, the MDP-CLLA model achieves best improvement by 6.7%, 13.95%, 22.26%, and 42.02% for 1000; 2000; 4000; and 8000 bits compared with the existing system and by 2.35%, 5.84%, 15.01%, for 1000; 2000; and 4000 bits compared with the CLLA model. By contrast, for 8000 bits, no improvement was noted in the MDP-CLLA model when compared with the CLLA model because they both have the same phase productivity. As a future work direction, this research could handle 5G network because it focuses on cross-layer technique and MDP that uses FSMC for modeling the wireless channel dynamic. In addition, the optimal packet size needed for each modulation stage and discussed in the result is not deployed within the LA models in this research. If this direction is considered, then an adaptive packet size can be considered as well apart from adapting the modulation considered in the proposed models. Hence, further enhancement in throughput is introduced.

## Data Availability

Not applicable. The results are based on real simulation data.

## Abbreviations

LA: Link adaptation
MS: Modulation scheme
SNR: Signal to noise ratio
CLLA: Cross-layer link adaptation
MDP-CLLA: Markov decision process over the CLLA
SISO: Single input single output
ANOVA: Analysis of variance
LTE: Long-term evolution
LTE-A: Long-term evolution-advanced
DSL: Digital subscriber line
DL: Downlink
QoS: Quality of service
MSDUs: MAC service data units
CSI: Channel state information
AMC: Adaptive modulation and coding
FER: Frame error rate
OFDMA: Orthogonal frequency-division multiple access
MC: Modulation coding
MANET: Mobile ad hoc networks
MDP: Markov decision process
OLLA: Outer loop link adaptation
MAB: Multi-armed bandits
ANN: Artificial neural network
MIMO: Multiple-input-multiple-output
UE: Unit equipment
CQI: Channel quality indicator
PDF: Probability density function
FSMC: Finite-state Markov channel
ASR: Adaptive switching SNR range
SPSS: Statistical package for the social sciences
SD: Standard deviation
